# Ionic Liquids as Antibacterial and Drug Delivery Agents: How Cationic Amphiphilic Structure Controls Morphology Changes in Lipid Bilayers and Penetration Mechanism

**DOI:** 10.1021/acs.jpcb.5c07740

**Published:** 2026-03-02

**Authors:** Ludmila Baldan do Rosario, Leticia Rafaella Dias, Andrea Paravani da Costa, Asdrubal Lozada-Blanco, Kalil Bernardino

**Affiliations:** † Laboratório de Química Computacional, Chemistry Department, Universidade Federal de São Carlos, Rod. Washington Luiz S/n, São Carlos 13565-905, Brazil; ‡ Chemistry Department, FFCLRP, University of São Paulo, Av. Bandeirantes 3900, Ribeirão Preto, SP 14040-901, Brazil

## Abstract

Ionic liquid-based technologies are promising both as antibacterial agents and in drug delivery, as they can improve drug solubility and capacity to bypass lipid bilayers while also taking advantage of ionic liquids’ physical properties, such as negligible vapor pressure and stability. In both applications, it is imperative to understand how the molecular structure of the ionic liquid determines its interaction with cellular membranes. In this work, molecular dynamics simulations with coarse-grained models were applied to study the penetration of eight ionic liquids based on the 1,3-dialkyl-imidazolium cation with different alkyl group sizes into DPPC bilayers from both dilute and concentrated aqueous solutions. Potential of mean force calculations were performed to evaluate the thermodynamics of cation penetration, and graph theory was used to characterize their nonhomogeneous distributions inside the bilayers. Distinct effects were noticed over the bilayer morphology: Cations with a single small or medium alkyl tail do not induce significant changes over the bilayer structure, while cations with a 16-carbon-atom chain are water-insoluble and, in concentrated solutions, are capable of partially removing lipid molecules. Incorporated cations with two medium-sized tails remain close to the water interface, reducing the interaction between lipids and decreasing the bilayer thickness, while cations with two long tails penetrate into the hydrophobic center of the bilayer and increase its thickness instead. As a consequence of the different interactions, two distinct mechanisms have been proposed for the drug delivery action of ionic liquids, depending on their water solubility and clustering tendency.

## Graphical abstract

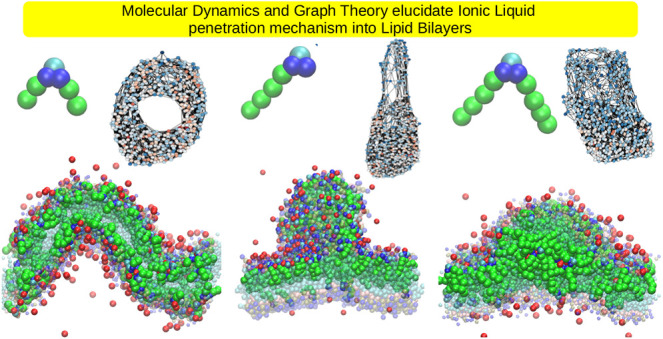

## 1 Introduction

Among molecules synthesized or discovered with potential pharmacological activity, many do not achieve clinical or even *in vitro* tests due to low water solubility or poor capacity to penetrate lipid bilayers.^1^ In this sense, ionic liquids (ILs)-based technologies emerged as a promising alternative to improve drug bioavailability.^2–4^ ILs are defined as salts with melting point lower than 100 °C, which is achieved by the presence of bulky, low symmetry, and flexible ions,^5,6^ with the cation usually derived from an organic molecule with amphiphilic character.^7^ Physical properties like low melting point, negligible vapor pressure, medium to high viscosity, and good affinity for both polar and apolar species make ILs especially promising in topical formulations,^8^ which reduce collateral effects related to the oral administration, but faces challenges regarding the losses due to evaporation and the difficult to penetrate into the stratum corneum, the outermost layer of the skin at which the lipid bilayers presents an efficient barrier against the penetration of substances into the organism.^9^ In order to improve drug bioavailability, ILs can be employed either as a solvent, a cosolvent, or as a surfactant that can encapsulate hydrophobic drugs as micelles or microemulsions, or the drug itself can be converted into the cation or the anion of an ionic liquid.^2–4^ In the latter, molecules with an acid group, like the nonsteroidal anti-inflammatory drugs ibuprofen^10,11^ and mefenamic acid,^12^ can be deprotonated and paired with an IL forming cation, largely improving water solubility and also the kinetics of penetration into lipid bilayers.

Amphiphilic ILs are also promising as antibacterial agents, being able to act against bacteria resistant to traditional antibiotics.^13,14^ Cetylpyridinium chloride is employed as an antiseptic in oral care products^15^ and quaternary ammonium chlorides with two long alkyl tails, like dimethyldidecyl ammonium chloride (DDAC), are employed as disinfectants.^16^ Bipyridium-based cations displayed high activity against resistant bacteria like *Pseudomonas aeruginosa*, with the ones with gemini-cations with two medium-sized alkyl tails (around 11 or 12 carbon atoms) being more efficient than the cations with a single tail.^17–19^ Comparing two biscationic compounds with different alkyl group sizes, 1,10-diundecyl-4,4′-bipyridinium dichloride presents at least 10 times lower minimum inhibitory concentration against *P. aeruginosa* and at least 100 times lower against *Escherichia coli* when compared with 1-undecyl-10-methyl-4,4′-bipyridinium dichloride.^17^ Biscationic ILs based on bis-imidazolium also displayed at least 10 times the efficiency of the ILs with corresponding monomeric cation against both Gram-positive and Gram-negative bacteria,^20^ indicating that the presence of multiple polar and apolar groups in the cation renders greater antimicrobial activity. Although the mechanism of action is probably related to changes induced by the amphiphilic cations over the bacterial lipid bilayer, the details at the molecular level have not been completely elucidated. A recent work used neutron scattering to show that increasing the cation alkyl group leads to improved lateral diffusion in lipid bilayers, which leads to bacterial death, but did not include ILs with multiple alkyl groups in the study.^21^

For both improving drugs’ bioavailability and use as antibacterial agent, it is imperative to understand, at the molecular level, how ILs interact with lipid bilayers, since this will control both their drug delivery capacity as well as their toxicity. Computer simulations proved to be powerful tools to elucidate ionic liquids structure and physical properties, being able not only to explain, but also to anticipate properties that were only confirmed by experiments afterward.^22^ Regarding the interaction between ILs and lipid bilayers, molecular dynamics simulations showed the spontaneous penetration of amphiphilic cations with the charged portion staying close to the phosphate groups of phospholipids,^23,24^ the increase in the bilayer roughness due to cation penetration,^25^ changes in the bilayer thickness and electrostatic potential due to IL incorporation,^26^ and how the interaction between ILs with membrane channel proteins affects water and sodium ions penetration.^27,28^ In the present work, molecular dynamics simulations were performed to study the effect of the cation alkyl group on the interaction and penetration into lipid bilayers and the morphological changes induced. Holding the tetrafluoroborate, BF_4_^–^, as the anion, eight different 1,3-dialkyl-imidazolium cations were studied by changing the size of the alkyl groups, including sets of liquids with only one and with two long alkyl chains.

## 2 Methods

### 2.1 Model System Preparation and Interaction Parameters

In order to verify the effect of the size and number of alkyl groups on the penetration of ionic liquids in lipid bilayers and the morphological changes associated, eight different ILs were studied by changing the size of the two alkyl groups bonded to the 1,3-dialkyl-imidazolium cation while holding the tetrafluoroborate (BF_4_^–^) as the anion. For the rest of this article, we will use the notation CmCn for the ILs where m and n are the number of carbon atoms in each linear alkyl group (Figure 1a–c). Two sets of liquids were studied, one with a single tail (C4C1, C8C1, C12C1, and C16C1) and the other with two tails of the same size (C4C4, C8C8, C12C12, C16C16) in the cation. ILs with the BF_4_^–^ anion present relatively low melting points (*T*_m_) when compared with similar ones with Cl^–^ anions. For instance, 1-dodecyl-3-methylimidazolium chloride melts only at 97 °C^29^ while the *T*_m_ of 1-dodecyl-3-methylimidazolium tetrafluoroborate (C12C1) is 32 °C, being liquid at physiological temperature, while 1-hexadecyl-3-methylimidazolium tetrafluoroborate (C16C1) melts at 57 °C.^30^

**Figure 1:**
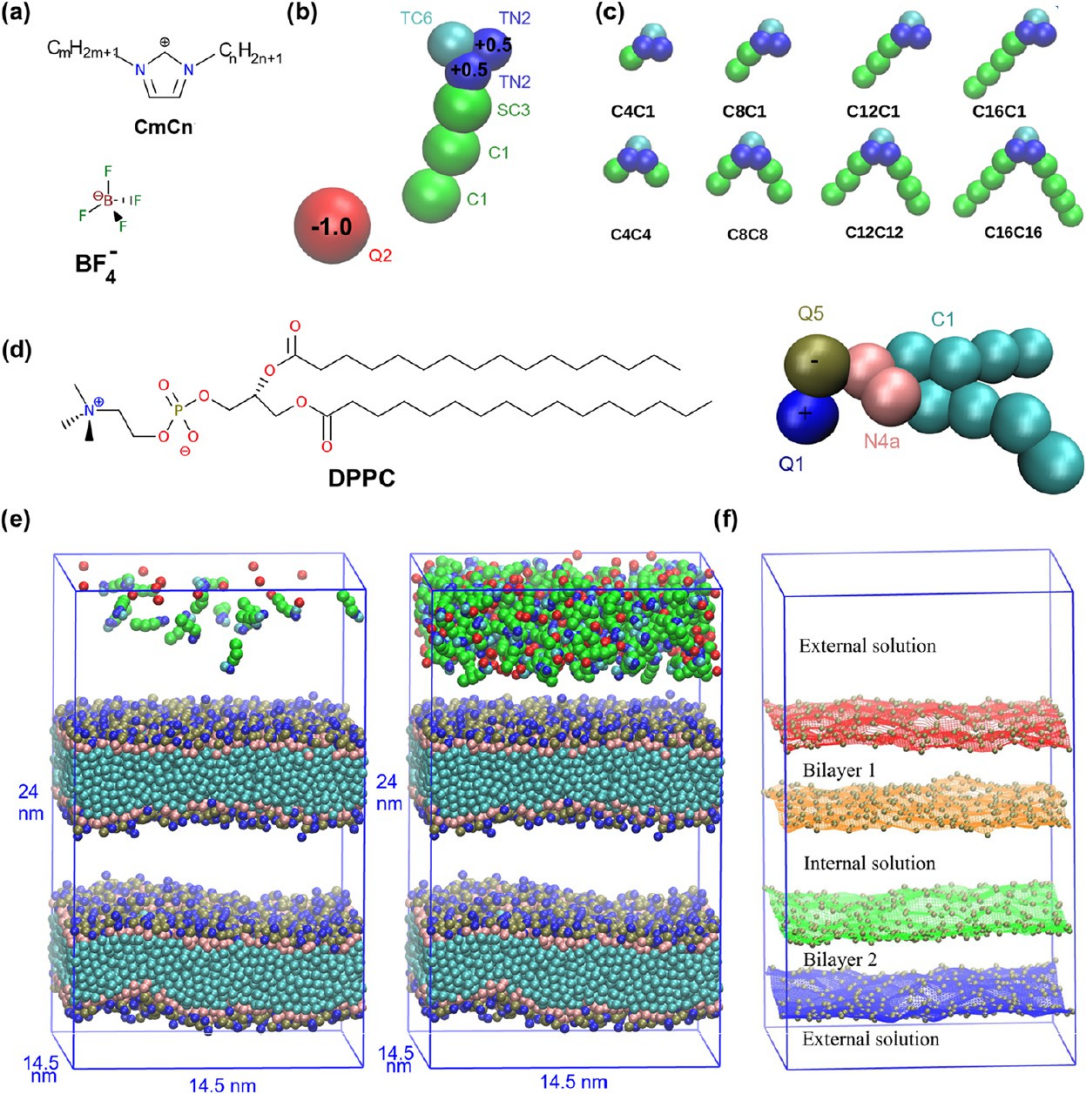
Description of the model systems. (a) Structural formulas of the cations and the anion of the IL. (b) Nonbonded parameters of Martini 3.0 force field and partial charges of ions, illustrated with C12C1 liquid. (c) Representation of all cations used in the simulations. (d) Structural formula and coarse-grained description of the DPPC (1,2-dipalmitoyl-*sn*-glycero-3-phosphocholine) lipid. (e) Initial structures of the model systems with 16 and 320 ion pairs of C12C1 IL. Water particles were hidden for better visualization. (f) Grids used to describe the bilayer interfaces generated by the interpolation of the phosphate groups (brown spheres) of DPPC in the initial structure and the corresponding volumes defined by the grids.

In order to enable the microsecond-scale simulations, the coarse-grained force field Martini 3.0 ^31,32^ was employed. In this force field, the tetrafluoroborate anion is described by a single hydrophilic and negatively charged interaction site Q2, the imidazolium ring of the cation is described by 3 interaction sites with the positive charge distributed between two sites of intermediate hydrophilicity (TN2), the first site of the alkyl tails is described by the hydrophobic SC3 parameter, and for each 4 additional carbon atoms, another hydrophobic C1 site is included (Figure 1b,c). The same force field parameters were used in our group to study the effect of the cation alkyl group over the stabilization of nanoparticles dispersed in ILs.^33,34^

As a simplified model of a biological membrane, two bilayers of the 1,2-dipalmitoyl-*sn*-glycero-3-phosphocholine lipid (DPPC) (structural formula and coarse-grained model in Figure 1d) were produced using the Packmol software^35^ with 640 DPPC molecules in each bilayer (1280 DPPC molecules in the model system) and the simulation boxes were filled with water particles while avoiding the insertion of water inside the hydrophobic core of the bilayers. The system was produced with 2 bilayers in order to reproduce an external solution, in which the ions of the IL were introduced by replacing water particles, and an internal solution, initially free of ionic liquid (Figure 1e). To reduce surface artifacts involved in simulating a small system, periodic boundary conditions were employed in the three dimensions, meaning that the simulation boxes of Figure 1e are connected to an infinite number of replicas. In model systems with only one bilayer, even if the ions were inserted only in one side of the box, due to the use of periodic boundary conditions in the perpendicular direction, the ions could be adsorbed at the two sides of the bilayer by moving into the periodic replica via aqueous solution, while in a real system the only way to have ions in the inner leaflet would be crossing the hydrophobic core of the bilayer. In the model with two bilayers, although the ions can be adsorbed in either of them, they will not be absorbed in the inner leaflet or appear in the internal solution unless they can cross the bilayer. To verify the concentration effect, two simulations were performed for each IL by introducing 16 ion pairs in the dilute systems and 320 in the concentrated ones (Figure 1e).

For potential of mean force (PMF) calculations, a smaller model system was produced with a single bilayer with 248 DPPC molecules, 5630 water particles, and one ion pair within a box with initial dimensions 9.0 × 9.0 × 12.6 nm (Figure S1). The umbrella sampling method with WHAM (weighted histogram analysis method)^36,37^ was employed by applying a harmonic potential to move the cation in the reaction coordinate ξ defined as the distance between the imidazolium group and the bilayer center. 46 sampling windows were performed for each ionic liquid to move the cation between ξ = 0.0 nm (center of the bilayer) and ξ = 4.5 nm (aqueous solution). 10 ns simulations were performed at each window and the force constant applied was 1000 kJ mol^–1^ nm^–2^, the same value used in previous works of our group.^38^

### 2.2 Software and Simulation Conditions

All the simulations were performed using Gromacs 2020 ^39,40^ version with *T* = 309 K maintained with V-rescale thermostat^41^ with τ_T_ = 1 ps and *P* = 1 bar coupled with Berendsen semiisotropic scheme^42^ with τ_P_ = 1 ps. A cutoff radius of 1.1 nm was used for all nonbonded interactions with the PME (particle-mesh Ewald)^43^ correction for long-range electrostatics and a shift potential to make the Lennard-Jones potential converge smoothly to zero between 0.9 and 1.1 nm. Also, a relative dielectric constant ε_r_ = 15 was employed to attenuate the coulomb interactions. An integration time step of 0.02 ps was employed for all simulations. VMD 1.9.3 ^44^ was used to render graphical representations from the trajectories.

### 2.3 Bilayer Morphology Analysis

In order to compute the concentration of ions in both internal and external aqueous solutions and inside the bilayer, taking the roughness and oscillations of the membranes into account, grids were generated by the interpolation of the atomic coordinates of the DPPC phosphate groups of each leaflet by using the Python LinearNDInterpolator library. Those grids will be taken as a description of the bilayer’s surface area and used to compute the bilayer thickness and the volumes of both the bilayers and the external and internal solutions (Figure 1f). This approach is similar to the one employed by SuAVE software,^45^ although we employed a homemade program for the grid generation and morphology analyses instead.

### 2.4 Graph-Based Analysis of Interaction Networks

To assess the structural organization of the system, graph-based approaches were employed to study the networks of interactions emerging from molecular dynamics simulations. In this representation, nodes were defined either by the DPPC phosphate groups forming the bilayer or by the cations present in the system, while edges were assigned according to the occurrence of specific interactions between these entities. These networks were then used to compute the graph measures degree and closeness centrality.^46^

All network calculations were performed using coor2graph, an in-house program developed by the authors, written in Fortran and parallelized for efficient computation along the molecular dynamics trajectory, and available at github.com/aslozada/coor2graph. Interaction data extracted from the molecular dynamics simulations were processed with coor2graph to construct and analyze the resulting networks using the *NetworkX* package.^47^ The program can be used to compute graph-based centrality measures, including degree, closeness, betweenness, katz, and eigenvector centrality, providing a quantitative characterization of the stability and communication pathways within the system. Further details of these calculations are given in the Supporting Information.

## 3 Results and Discussion

### 3.1 Thermodynamics of Cation Penetration into the Lipid Bilayer

In order to characterize the thermodynamic tendency of the different cations to be incorporated into the lipid bilayer, the potential of mean force (PMF) was computed to bring a single cation from aqueous solution to the middle of the DPPC bilayer (ξ = 0 nm). The transference from water to the bilayer is favorable for every cation considered, with the global minimum of the PMF located around ξ = 1.5 nm, which is close to the bilayer/water interface taken as the position of the lipid phosphate groups that displays a density maximum at ξ = 2.0 nm (vertical dashed line in Figure 2a). As expected, the increase of the alkyl group size leads to more negative values for the transference free energy, with a nearly linear variation with the number n of carbon atoms in both CnC1 and CnCn cations. The linear regression for CnC1 cations resulted in a linear relation Δ*G* = −3.2*n* – 10.6 with *R* ^2^ = 0.98, while for CnCn, Δ*G* = −5.8*n* – 4.0 with *R* ^2^ = 0.97 (linear regressions displayed by dotted orange lines in Figure 2c). Thus, the variation in the free energy for each CH_2_ group is −3.2 kJ/mol in the cations with a single chain and −2.9 kJ/mol in the cations with two alkyl chains. The smaller variation in the two-tailed is due to the steric hindrance one chain imposes over the other, reducing the exposed area to water of the groups at each tail. Those values lie between the typical contributions of 3.8 kJ/mol of hydrophobic energy per CH_2_ in linear alkanes when separating phase from water and 1.8 to 2.8 kJ/mol per CH_2_ group when a linear surfactant self-assembles into micelles.^48^ Those values also agree with the PMF calculations performed using atomistic force fields by Yoo et al. for the penetration of [C4C1]^+^ and [C12C1]^+^ cations into POPC bilayers.^21^

**Figure 2:**
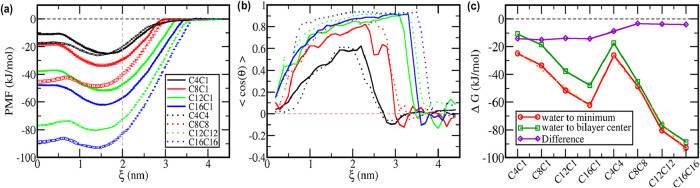
Thermodynamics of cation penetration into lipid bilayer. (a) Potential of mean force (PMF) for the penetration of a single cation into the DPPC bilayer, with ξ = 0 being the bilayer center and the vertical dashed line stands for the maximum of the DPPC group phosphate density. Error bars computed by bootstrap method by dividing the data at each window in five sets and computing the standard deviation of the resulting PMFs. (b) Average orientation of the cation along the PMF, with θ being the angle between the vector from the center of geometry of hydrophobic sites to the center of geometry of hydrophilic sites of the cation and the vector normal to the bilayer. A running average at every 4 points was performed to reduce the noise. (c) Free energy variation to move the cation from aqueous solution (ξ = 4.5 nm) to the corresponding PMF global minimum inside the bilayer and to the bilayer center and the difference between both values. Orange dotted lines are the linear regressions of Δ*G* values for moving from water to the PMF minimum with the size n of the alkyl chain(s).

The orientation of the cations along the PMF was evaluated by computing the angle θ between the vector going from the center of geometry of the hydrophobic sites (the tail) to the center of geometry of the hydrophilic sites (imidazolium ring) and the vector normal to the bilayer. Hence, positive cos­(θ) indicates a preference to orient the imidazolium ring away from the center of the bilayer (Figure 2b). At the PMF minimum, all the cations are preferentially oriented with the polar portion pointing toward water and the apolar portion close to the bilayer center as expected, but this effect is less significant for the C4C1 and C4C4 cations. No significant difference is noticed between cations with one and with two alkyl groups regarding the orientation inside the bilayer. Far from the bilayer, no preferential orientation is expected, and the average values of cos­(θ) only fluctuate around zero. However, the cations with longer alkyl groups exhibit preferential orientation at longer distances from the bilayer since the longer tail can still interact with the bilayer to reduce exposition of hydrophobic sites to water. Interestingly, the cations also display random orientation close to the center of the bilayer since in this situation the imidazolium is buried in the hydrophobic core of the bilayer and do not interact significantly with water or polar portions of the lipids.

Besides the large thermodynamic tendency to be incorporated into the lipid bilayers, all cations displayed a barrier to cross the hydrophobic center. This barrier, however, is more significant for cations with a single alkyl chain. The free energy difference between the global minimum of the PMF and the bilayer center (ξ = 0) stands for 14 ± 1 kJ/mol for all cations with a single alkyl group, being independent of the alkyl chain size. However, when a second alkyl chain is introduced, this difference decreases to 4 ± 1 kJ/mol for all two-tailed cations studied except the C4C4. Therefore, the free energy barrier for bypassing the bilayer center does not change appreciably with the alkyl group length, but is largely reduced when a second long alkyl group is introduced. This difference is due to the steric hindrance the second chain introduces over the cation charged ring, which diminish its interaction to polar or charged species like the favorable interaction with the phosphate groups of the DPPC lipids (vertical dashed line in Figure 2a indicates the position of the phosphate groups, radial distribution function between cation imidazolium ring and DPPC phosphate given in Figure S16). The small free energy difference between the interfacial region and the hydrophobic core also implies that the two-tailed cations should be more distributed inside the bilayer, as is noticed by both the visual inspection of the trajectories and the density profiles in systems with several ion pairs, which will be discussed in the following section.

### 3.2 Spontaneous Cation Penetration and Induced Changes in Membrane Morphology

In concentrated solutions, the solubility and aggregation tendency of the ILs determine the dynamics of incorporation into the lipid bilayers. The ones with small alkyl groups (C4C1 and C4C4) present high water solubility and small tendency to cluster (Figure 3a). Therefore, each cation diffuses separately and the penetration into the bilayers happens individually. On the other hand, C8C1 and C12C1 cations aggregate, forming micelles in aqueous solution and the clusters diffuse and are incorporated into the bilayers (Figure 3b). In both cases, nearly the same number of cations is incorporated in each of the two bilayers. A different behavior was noticed for the liquids with two large (*n* > 4) alkyl tails. Those liquids present a low water solubility, and even starting with a homogeneous distribution, a phase separation quickly takes place, forming nanodroplets before a significant incorporation into the bilayers happens. As a consequence, the incorporation happens by the diffusion of the whole droplet toward the membrane and, once the droplet touches the bilayer, a strong perturbation is induced upon the lipids as the ions migrate together to the bilayer and all or almost all cations are incorporated in the same bilayer (Figures 3 and 4). In dilute IL solutions, the cations diffuse independently into the lipid bilayers in all systems except in C16C16, where a small cluster was formed even in the dilute system (Figures S2 and S3). A rather unique case happened for the 1-hexadecyl-3-methylimidazolium tetrafluoroborate, C16C1: As the liquids with two alkyl tails, this one undergoes phase separation before incorporation in the bilayer; however, this is probably due to the tendency of this liquid to display a smectic liquid crystal phase (Figure S4), it forms a disk-like instead of a spherical droplet and, upon the contact with the bilayer, some cations diffuse into it but others remains in a semidisk structure and removes DPPC molecules from the bilayer, a structure that remains even after 1000 ns (Figure 4).

**Figure 3:**
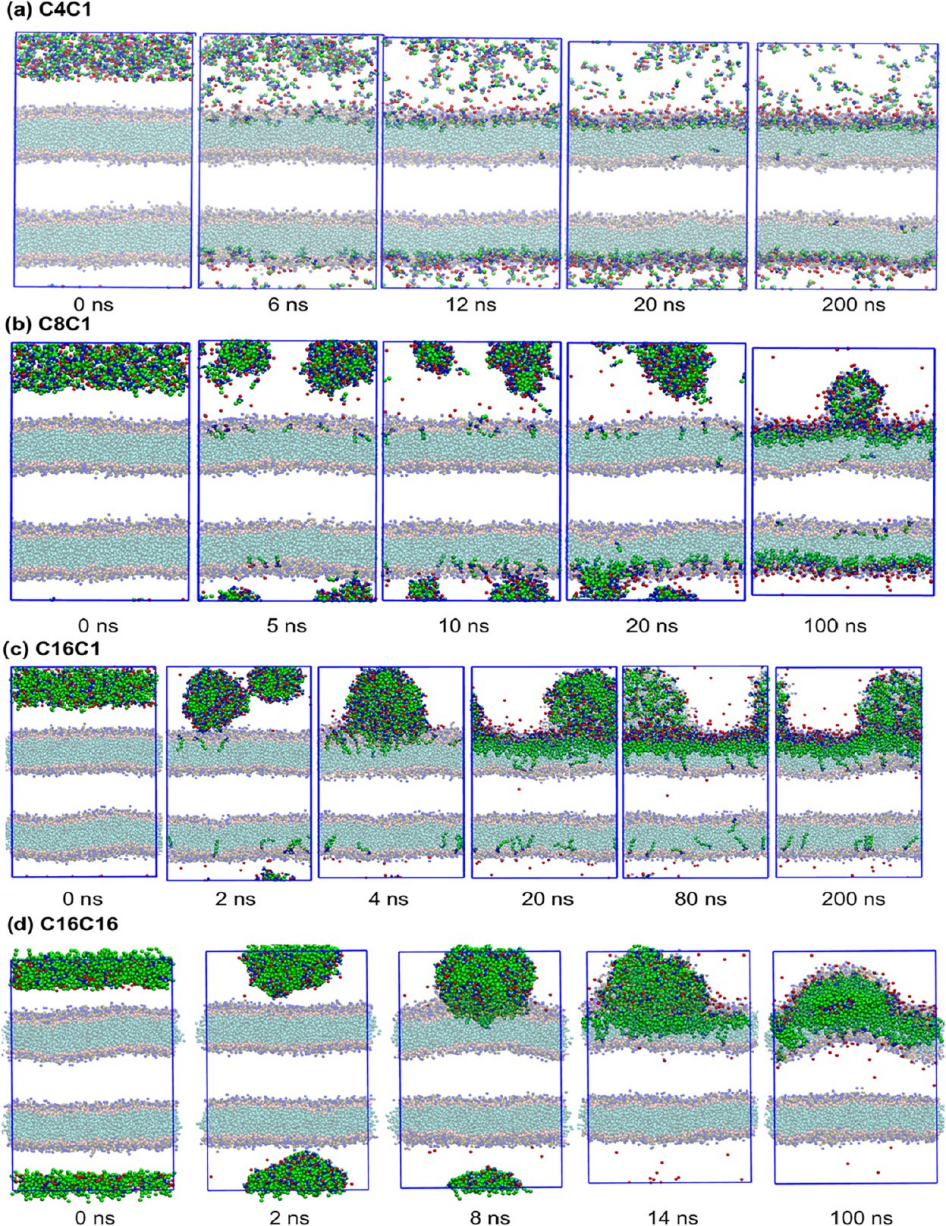
Dynamics of ionic liquid penetration into the bilayers. Selected structures alongside the relaxation of the systems with 320 ion pairs of the ILs: (a) C4C1, (b) C8C1, (c) C16C1, and (d) C16C16. Water was hidden for better visualization.

**Figure 4:**
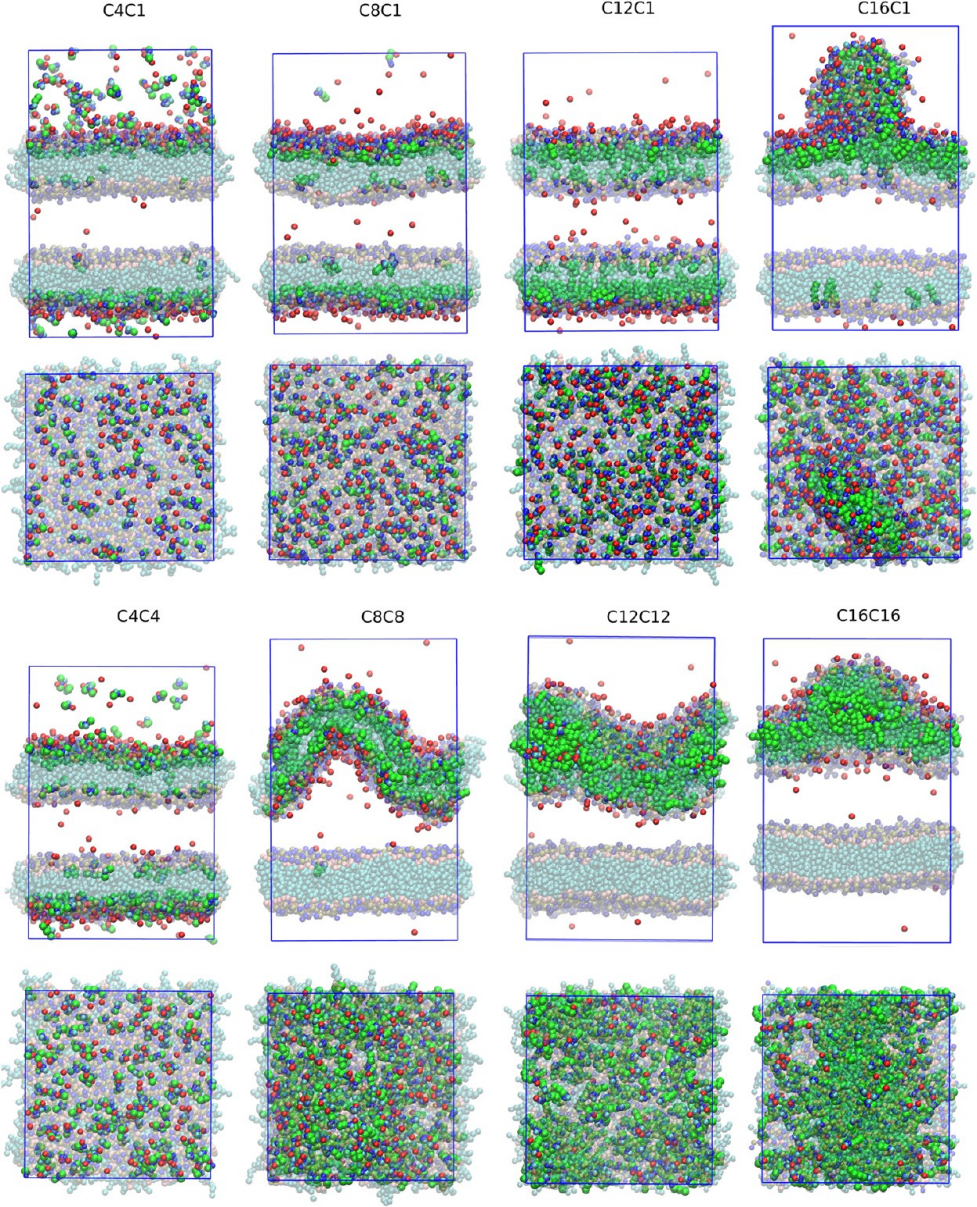
Ionic liquids distribution inside the lipid bilayer. Lateral and top view from the final structures (1000 ns) from molecular dynamics simulations of each ionic liquid interacting with the DPPC bilayer in the models with 320 ion pairs. DPPC lipids are displayed as transparent van der Waals spheres, while cations and anions from the ILs are displayed as opaque spheres. Water was hidden for better visualization. In the top views, only the bilayer with the highest cation density is shown, with the cations and anions that are in contact with at least one DPPC molecule from the bilayer.

The final structures after 1000 ns simulation for the concentrated solutions are presented in Figure 4, while those for the dilute ones are presented in Figure S3. In the systems with 16 ion pairs, all the cations were incorporated into the bilayers at the final structure. On the other hand, in those with 320 ion pairs, some C4C1, C8C1, and C4C4 cations remained in aqueous solution. The lateral view of the final structures and the density profiles (Figure 5 for cation imidazolium group density in every system, and Figures S5 to S12 for ions and DPPC densities in each system separately) reveal differences in the cation distribution along the distance ξ from the bilayer center: For all liquids except C12C12 and C16C16, most of the cations remained adsorbed at the side of the bilayer facing the external solution (negative ξ in Figures 5 and S5 to S12). This imbalance between the number of cations at each side of the bilayer is a consequence of the free energy barrier that cations need to overcome in order to cross to the internal layer, as discussed in the previous section. This barrier is smaller for the cations with two long alkyl groups; hence, those display larger densities at the internal layer (positive ξ) when compared to the corresponding ones with only one alkyl chain.

**Figure 5:**
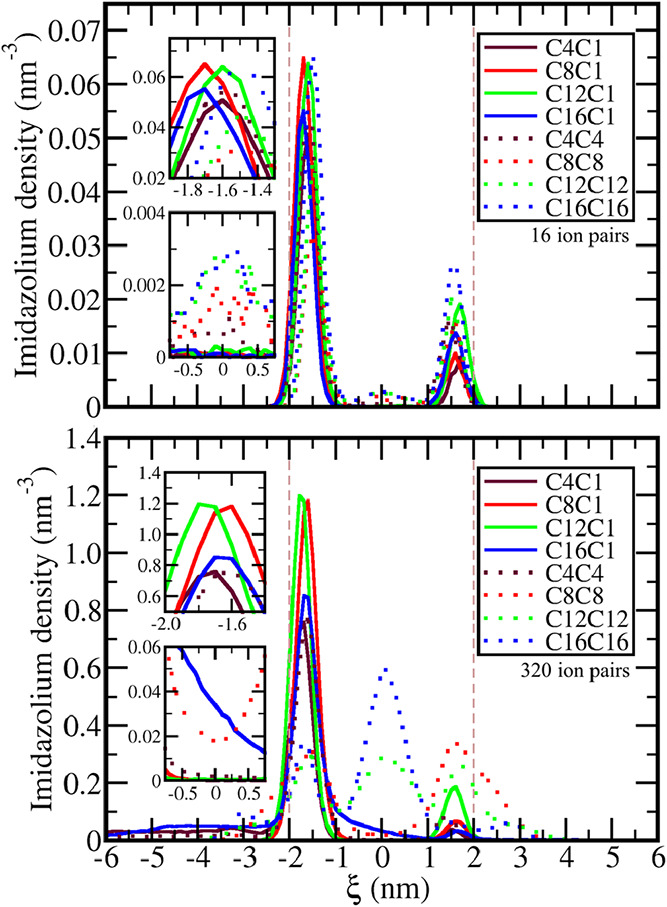
Cation distribution. Density profile for cation imidazolium rings center of geometry distribution in relation to the distance ξ from the bilayer center in the model systems with 16 (top) and with 320 ion pairs (bottom). All curves shown correspond to the bilayer with the larger number of cations of the IL incorporated, and negative ξ corresponds to the side of the external solution, while positive ξ corresponds to one of internal solution. Vertical dashed lines indicate the position of the maximum of DPPC phosphate group in both sides of the bilayer in the system with C8C1 ionic liquid. Insets zoom over the maxima and around the center of the bilayer (ξ = 0).

The presence of a local minimum at the center of the bilayer (ξ = 0) in the PMFs of the cations with two alkyl groups also leads to a larger cation density at the hydrophobic core of the bilayers when compared with the cations with a single chain in both concentrations studied. In the case of the strongly hydrophobic cations C12C12 and C16C16, this trend became even stronger in the systems with 320 ion pairs, leading to density maxima in the middle of the bilayer. For C12C12 (green dotted curve in Figure 5), the density at the center of the bilayer is nearly the same as at both interfaces, indicating that this cation can be either incorporated at the interface between the hydrophilic and hydrophobic portions of the bilayer as well as migrate to the hydrophobic center. Increasing the tails leads C16C16 to prefer the hydrophobic core of the DPPC bilayer instead of the interfacial region. Those two ILs also show a nonhomogeneous distribution of the cations inside the bilayer in the concentrated systems, as noticed by the top views in Figure 4. In the case of C16C16, we can clearly notice a phase separation inside the bilayer with the ions concentrated in the central area of the representation.

Besides the visual inspection, the segregation of the ionic liquid inside the bilayer can also be noticed by the radial distribution function between alkyl groups (Figure S15) and charged groups (Figure S18) of the ionic liquid, both displaying stronger long-range correlations for the liquids with two longer tails except C4C4. Even in the dilute solutions, a stronger tendency of cation–cation interaction is noticed inside the bilayers for the ones with two alkyl groups (structures in Figure S3 and radial distribution function in Figures S15).

Another difference revealed by the cation imidazolium group density profiles is the relative optimal position of the cations at the membrane/water interface: Except for C4C1 and C4C4, the position of the density peak in the dilute systems (16 ion pairs) is slightly shifted closer to the bilayer center when a second long alkyl group is introduced, which correlates with the shift in the minimum of the potential of mean force (Figure 2). This could be attributed to two distinct effects: an increase in the hydrophobic effect and the larger steric hindrance over the charged imidazolium ring upon the introduction of the second tail. However, since no systematic difference in both the density maximum and PMF minimum positions was noticed due to the increase of the tail from C8C1 to C16C1, the steric hindrance over the polar region must be the major effect regarding the position of the imidazolium ring. This conclusion is also corroborated by the radial distribution function between the cation head and DPPC phosphate group (Figure S16), which is not affected by the size of the alkyl group in dilute systems but shows a significant reduction when the second tail is introduced. The shift in the cation location, although small, reduces the exposition of the cation to water and to other hydrophilic species that may be present at the bilayer surface, including carbohydrates and surface proteins, implying that adsorbed cations may affect in different ways processes of cellular recognition. In the concentrated systems, although this trend is also noticed for C4C4 and C8C8, the interpretation of the effect of the number of tails over the density profile becomes less straightforward due to the changes over the bilayer morphology as will be discussed in the following.

Three different metrics were used to quantify the changes induced over the bilayer morphology due to the interaction with the ILs: The area per lipid, the volume per lipid, and the bilayer thickness. The results are presented only for the concentrated systems (320 ion pairs) since in the dilute ones, those metrics remained essentially unchanged when compared to the reference values of the same bilayers without the IL (Figure 6). We will call “Bilayer 1” the one with the largest IL cation concentration in the corresponding system and “Bilayer 2” the one with the smallest concentration and, in the case of the surface area, the “External” refers to the side facing the external solution (where the IL is initially introduced, red and blue grids in Figure 1e) and “Internal” to the side facing the internal solution (green and orange grids in Figure 1e). In the case of the volume, the average between the two bilayers were also introduced to facilitated the evaluation of the effect of the alkyl group size despite the fact in some cases (C4C1, C8C1, C12C1, and C4C4) the penetration happened evenly in the two bilayers while in the ones that displays a segregation first (C16C1, C8C8, C12C12, and C16C16) almost all the cations were incorporated in a single bilayer. When the average between the two bilayers was taken into account, a linear increase of the bilayer volume with the size of the tails is noticed.

**Figure 6:**
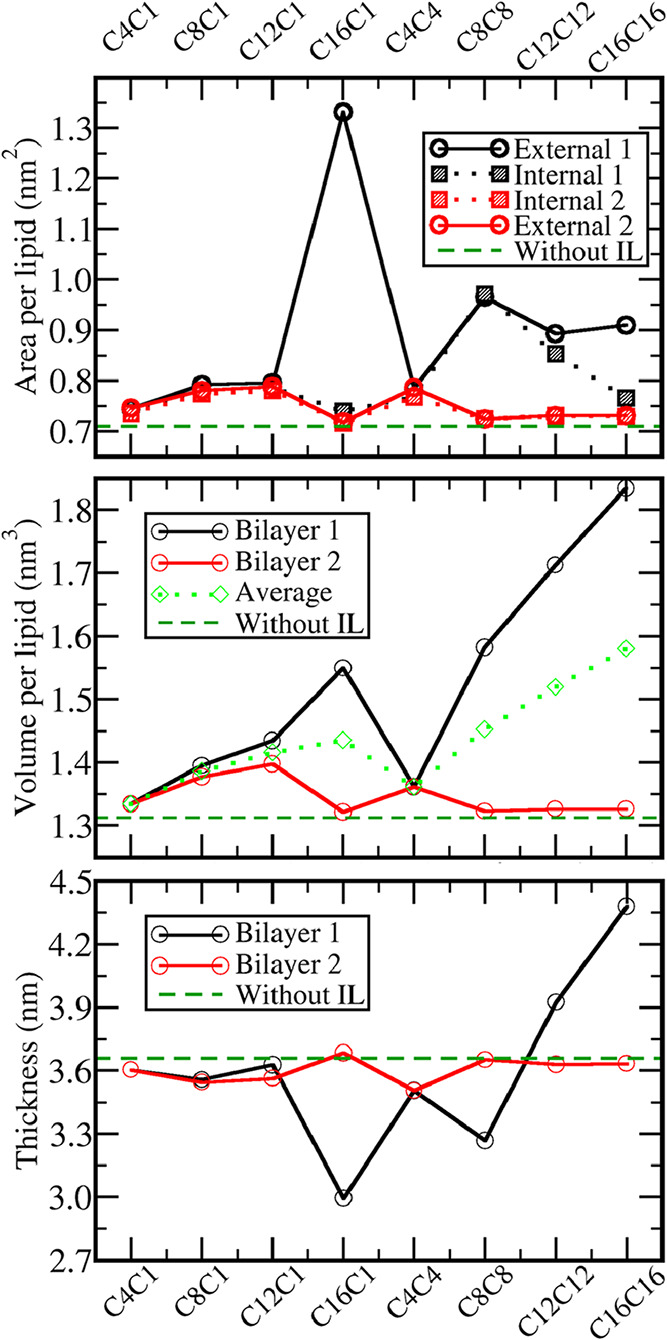
Bilayer morphology. Top: Average surface area of the sides of the bilayer facing the internal and external solution per lipid molecule in each layer. Middle: Average volume of each bilayer divided by the number of lipid molecules in the bilayer. Bottom: Average bilayer thickness. Green dashed lines correspond to the result in the absence of any ionic liquid. Bilayer 1 refers to the one with the high cation density after relaxation.

The penetration of the liquids with two tails results in larger increases both in the bilayer area and volume when compared with the corresponding ones with a single tail, except for the area in the exotic case of C16C1. The addition of a second tail on the opposite side of the imidazolium ring makes the cation alkyl groups unable to be both arranged perpendicular to the lipid alkyl groups; otherwise, one of the tails would be pointing to aqueous solution. Two tails pointing in opposite directions lead to a larger distance between DPPC molecules and a larger area variation (Figure 6). Regarding the cations with 2 tails, the larger area and, consequently, the smaller thickness are found for C8C8. This seems to be counterintuitive since one may expect the cations with longer tails to induce larger effects. However, both C12C12 and C16C16 tend to penetrate inside the hydrophobic center of the bilayer as discussed previously, while C8C8 still remains mainly at the interface. This results in C12C12 and C16C16 actually increasing the bilayer thickness instead of decreasing and presenting smaller effects over the surface area in concentrated solutions.

Those effects explain the efficiency of different cations as antibacterial agents. For instance, for 4,4′-dialkyl-bipydinium cations, the smaller inhibitory concentration is found for the ones with two alkyl tails with around 10 or 11 carbon atoms each.^17,18^ A single alkyl tail enables a perpendicular packing with DPPC molecules, which results in smaller effects over the bilayer morphology than when two medium-sized tails are present. Hence, the cations with two medium-sized tails in opposite sides result in larger area increases and also a larger thickness decrease of the membrane, which may facilitate its rupture and the penetration or the loss of other substances inside the cell. On the other side, the ones with longer tails present water solubility issues, but even when properly dispersed, their deeper penetration into the hydrophobic core results in a relatively unchanged membrane surface.

In the case of C16C1, the exotic structure of an IL semidisk adsorbed at the bilayer (Figures 3 and 4) renders a much larger area increase than observed in both C16C16 and in the other ILs with a single tail, but only over the external area of the bilayer in contact with the disk, indicating that, despite the huge impact over the structure of the external DPPC layer, the internal layer remains relatively unchanged. Although the structure formed in C16C16 is different from that of C16C1, with the IL nanodroplet penetrating inside the hydrophobic core of the bilayer, the effect over the bilayer surface area is also asymmetric, with a larger increase over the external area than over the internal area. A similar trend is also noticed for the area for C12C12, although with smaller intensity than for C16C16 as the tendency of penetration and phase separation inside the bilayer is smaller.

The differences in the cation penetration also affect the interaction involving DPPC molecules in comparison to the IL-free bilayer (Figure 7). In dilute IL solutions, where each cation acts nearly independently of the others, the changes with the number of carbon atoms in the alkyl tails are linear for every energy component involving DPPC molecules (Figure 7 top). As the cation alkyl groups increase, the interaction energy between DPPC and the cations becomes more negative as there are more hydrophobic interaction sites in each cation in contact with DPPC tails. Concomitantly, the contact between DPPC molecules is reduced by the incorporation of the cation, rendering positive Δ*E* values, which increases as more interaction sites per cation are introduced into the bilayer. Notice, however, that the gain with the interaction with the cation suppresses the loss in the DPPC–DPPC interaction energy. Besides smaller intensity, we also notice a systematic variation in the DPPC-water interaction, showing that the penetration of the cations increases the exposed area of lipid molecules to water, with the effect also becoming more important as the alkyl groups increase in the dilute IL solutions.

**Figure 7:**
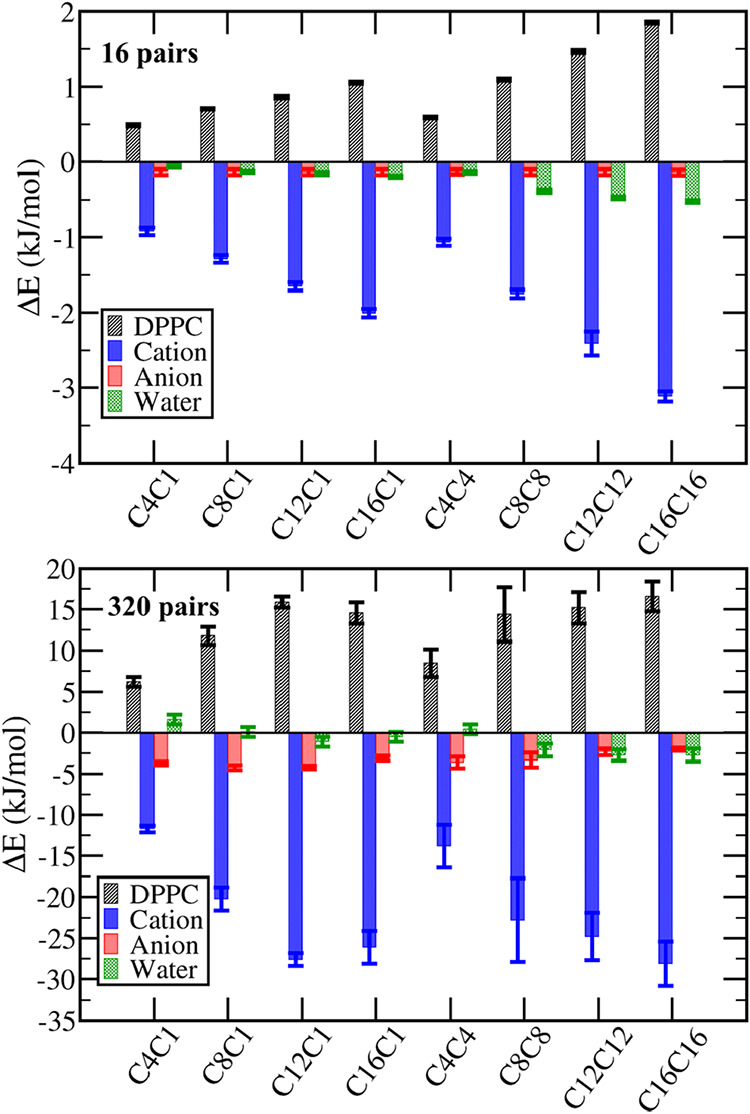
Potential energy changes upon ionic liquid penetration. Variation of the average interaction energy between DPPC molecules with other DPPC molecules, ions of the ionic liquid, and water per DPPC molecule due to the presence of the ionic liquids, with the corresponding standard deviation displayed as error bars. Top: Systems with 16 ion pairs, bottom: systems with 320 ion pairs.

Naturally, larger changes in the potential energy are expected as the concentration of the IL increases, and more complex variations were noticed for the concentrated IL solutions (Figure 7, bottom), as the cooperative effects between the ions become significant. From C4C1 to C8C1, from C8C1 to C12C1 and from C4C4 to C8C8, we notice that the DPPC-cation interaction energy becomes more negative with the increase of the alkyl chain as in the dilute system, but from C12C1 to C16C1 and from C8C8 to C12C12 there is no change inside the standard deviation due to the tendency of C16C1, C12C12 and C16C16 to undergo a phase separation instead of an homogeneous distribution inside the bilayer, rendering a saturation of the lipid-cation interaction besides the increase of interaction sites per cation. This also reflects in no significant change in the DPPC–DPPC interaction when increasing the tail size beyond 12 carbons in the CnC1 ILs or beyond 8 carbons in CnCn ILs in concentrated solutions.

The Δ*E* for the DPPC-water interaction, although small, shows interesting variations, being positive for both C4C1 and C4C4, nearly zero for C8C1, and negative for the other ILs. This is due to two opposite effects: while the presence of the cation itself increases the exposed area of the lipid, resulting in a negative Δ*E* with water as noticed in the dilute solutions, the increase in the cation density also leads to stronger anion adsorption over the membrane surface, as noticed by the significant more negative DPPC-BF_4_^–^ interaction in the systems with 320 ion pairs and in the radial distribution functions (Figure S17). The larger anion adsorption reduces the water-DPPC contacts, leading to a positive Δ*E* in most of the concentrated systems. The cations with small tail groups, however, result in smaller anion penetration, as will be discussed in Section 3.4. Hence, for C4C1 and C4C4, this effect is smaller than for the other ILs, and the increase in the exposed DPPC area due to cation adsorption is the dominant effect.

The dynamics of the cations and lipid molecules were evaluated by the diffusion coefficient (Figure 8). It is important to notice, however, that coarse-grained force fields usually lead to faster dynamics, overestimating the diffusion coefficients. The diffusion coefficient reported in atomistic simulations for DPPC at bilayers at 305 K performed by Kong et al. is *ca*. 1.2 × 10^–7^ cm^2^/s,^49^ while the diffusion coefficient computed in our simulations in the absence of the IL at 310 K stands at 6.9 × 10^–7^ cm^2^/s. Hence, the diffusion coefficient data presented in the rest of this section should be regarded as qualitative data only. In both dilute and concentrated solutions, the diffusion coefficient of the single-tail cations decreases with the increase of the alkyl group between C4 and C12, which could be expected due to the increase of both the molar mass and the van der Waals interactions between the cation and DPPC tails with increasing alkyl groups. However, no significant change is noticed between C12C1 and C16C1 in dilute solutions, and an increase is observed in concentrated solutions, which is due to the exotic structure in the C16C1 system that leads to a decrease in the cation-lipid interaction. On the other hand, the cations with two tails display only a decrease between C4C4 and C8C8, with the further increase of the alkyl group resulting in no significant effect on the cation diffusion in dilute solutions. In the concentrated system, the diffusion of C16C16 decreases due to the phase separation noticed inside the bilayer. A word of caution is needed when considering the diffusion coefficients for C4C1 and C4C4: Since those cations are weakly bonded to the bilayer, exchanges between the bilayer and water are frequent, and as the cations in water display faster dynamics, this leads to larger values for the diffusion coefficients of those two cations and also explains the difference between concentrated and dilute solutions for those two liquids: The larger values for concentrated solutions are due to a larger fraction of cations in aqueous solution in the concentrated system. Besides those two systems and C16C1 and C16C16, at which different kinds of phase separation were noticed, the IL concentration in the bilayer does not affect cation diffusion in our models.

**Figure 8:**
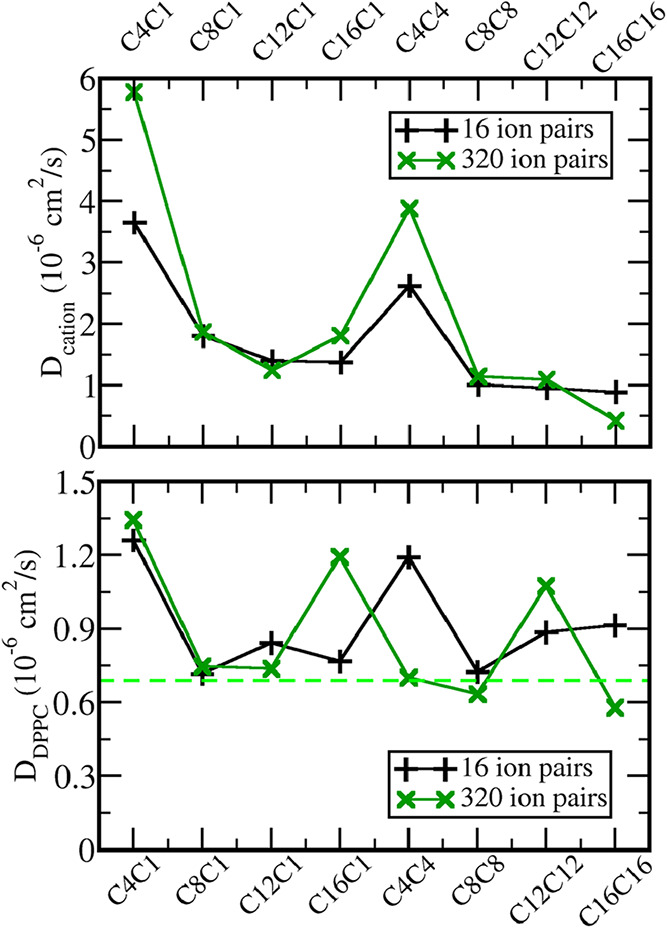
Molecular dynamics in the bilayers. Diffusion coefficient D of the cations (top) and of lipid molecules (bottom) for dilute (black curve) and concentrated (dark green curve) IL solutions. The dashed green line in the bottom panel corresponds to the DPPC diffusion coefficient within the same bilayers but in the absence of ionic liquid.

The effect of the cation on the diffusion coefficient of the lipid is complex. The C4C1 leads to an 100% increase in the lipid diffusion, while the C8C1, C12C1, and C16C1 results in no increase or only small increases in dilute solutions All cations leads to a decrease in the DPPC–DPPC interaction, as noticed before (Figure 7), but larger alkyl group results in a small van der Waals interaction with DPPC tails (radial distribution functions between tails in Figure S14 of Supporting Information file). Those effects may cancel each other, leading to no significant effect of the C8C1, C12C1, and C16C1 over the lipid diffusion in dilute solutions. Also, the larger orientation freedom of C4C1 and C4C4 compared to the other cations (Figure 2b) may also contribute for a faster diffusion of both the cation and DPPC. On concentrated solutions, however, C16C1 significantly breaks the contacts between lipid tails, leading to a significant increase in the diffusion (more details in the next section). C12C12 and C16C16 lead both to significant increases in lipid diffusion in dilute solutions, but while C12C12 also increases the diffusion in concentrated solutions, C16C16 leads to a decrease in the concentrated solutions. This strange behavior is also due to the phase separation noticed for C16C16, with the IL concentrated as a nanodroplet in the hydrophobic core of the bilayer. This is the opposite of C16C1, which also displays a phase separation but with the IL in the external side of the bilayer. The different effects of those two ILs on the lipid arrangement will be discussed in detail in the next section, using graph theory (GT). In summary, the size and number of alkyl groups lead to nonmonotonic changes in both the thickness of the bilayer and the dynamics of lipid molecules, with significant concentration effects in the systems that display phase separation.

### 3.3 Graph Theory Describes Cation Nonhomogeneous Distributions and Perturbations in the Lipid Bilayers

Graph theory (GT) is an interesting and powerful tool to characterize aggregation patterns and phase transitions in soft matter systems as discussed in a previous work.^50^ GT reduces the amount of information from the MD trajectories by retaining only information regarding the contact between molecules or ions, each molecule or ion being described as a node in the graph, with an edge introduced between the corresponding nodes if the molecules are in contact. As in our previous work,^50^ we will consider here only the contacts between the hydrophobic tails of the molecules since they provide the driving force for either the lipid bilayer formation or amphiphilic cation penetration. Hence, two DPPC molecules or two cations A and B are considered in contact with each other if any tail site of A is in the first shell of any tail site of B (defined by the radial distribution function first minimum at 0.7 nm, Figures S13 to S15). Two different properties were computed using GT: The degree, which corresponds essentially to the number of molecules each one is in contact with, and the closeness centrality, which measures how close each molecule is to the others in terms of the connections (edges) of the network. While the degree is a local property, the closeness centrality is a long-range one.^46,47^ More details about their definitions with examples are given in the Supporting Information file.

The graphs describing the cation–cation contacts in the final structures of the concentrated solutions (Figure 9 top) show that in the C4C1, C8C1, C12C1, and C4C4 systems, most of the cations remain isolated from each other. Still, larger networks are noticed as the alkyl groups increase. In C8C8, most cations are connected to other cations, but a significant number of independent cations remain and the connected cations form sparse small or medium-sized networks as they remain mostly at the bilayer/water interface. On the other hand, in C12C12 and, especially, C16C16, the IL penetrates the interior of the bilayer and remains concentrated in smaller regions, rendering in both cases a single large and more compact network, as noticed by larger values of both the degree and closeness centrality (Figure 9, bottom, and Table 1) while some cations still remain dispersed in the bilayer or form small clusters. The histograms of both degree and closeness centrality testify the phase separation of those ILs inside the bilayers by the presence of two populations, being the one with small values corresponding to the cations dispersed in the DPPC and the population with larger degree or larger closeness centrality corresponding to the IL phase inside the bilayer.

**Figure 9:**
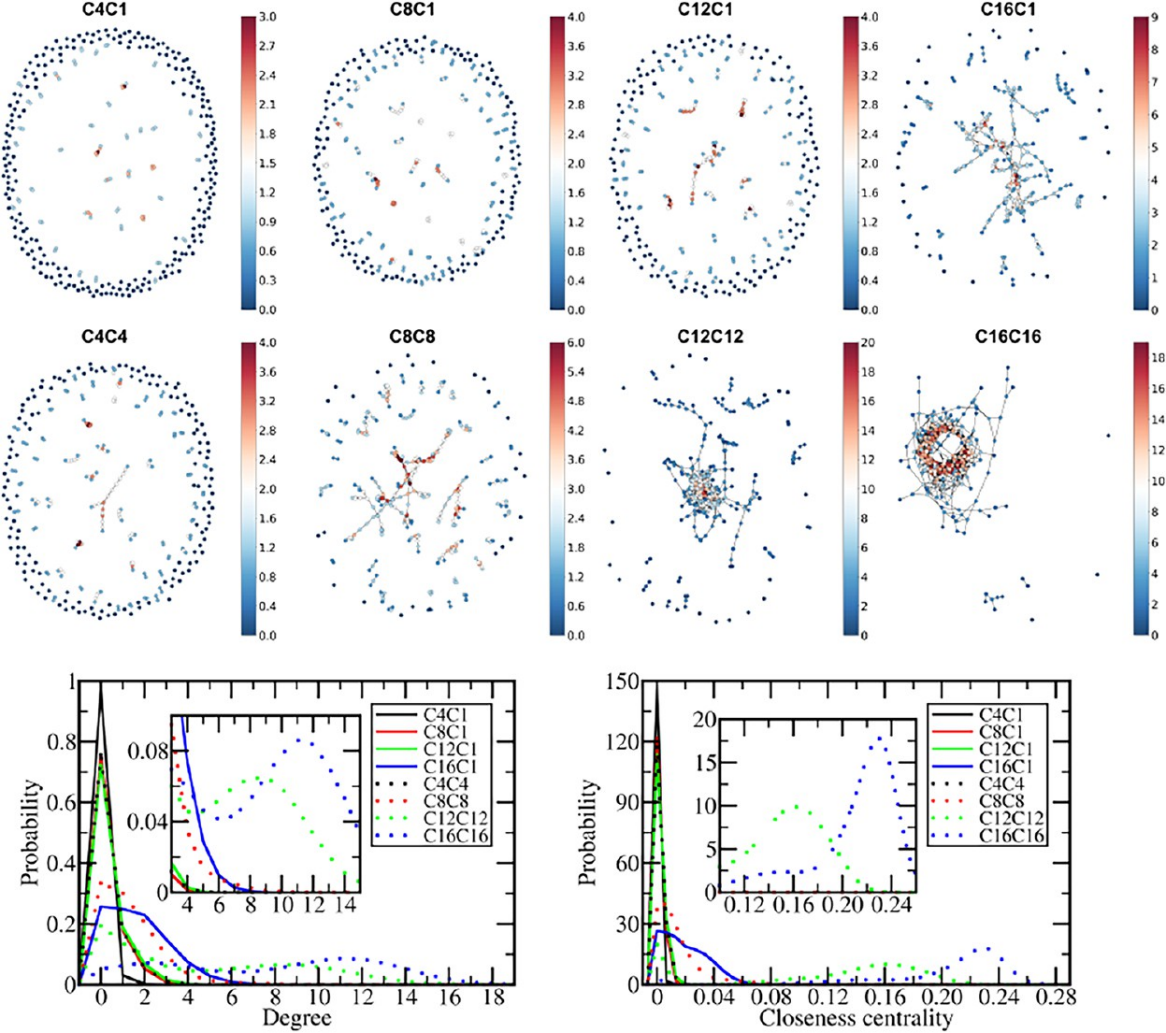
Graph theory description of the cations network. Top: Graphs representing the connection network between cation tails in the final structure of each system with 320 ion pairs colored based on the degree values. Bottom: Histograms displaying the distribution of degree and closeness centrality values for the cation network along the final 200 ns of the simulations in the systems with 320 ion pairs. Insets zoom over larger values of the degree or closeness centrality.

**Table 1:** Average Values of Degree and Closeness Centrality of Cation and DPPC Networks in Concentrated Systems

IL	C4C1	C8C1	C12C1	C16C1	C4C4	C8C8	C12C12	C16C16
degree cation	0.283	0.931	0.999	2.592	0.916	2.231	5.777	8.849
degree DPPC	14.333	13.897	13.387	13.122	14.202	13.401	12.700	12.165
closeness cation	0.001	0.004	0.004	0.022	0.004	0.017	0.123	0.208
closeness DPPC	0.113	0.110	0.108	0.106	0.112	0.107	0.105	0.103

GT also captures the unique behavior of C16C1, with most of the cations remaining on the dike-like structure and forming a large network; however, this network is more sparse, with fewer cation–cation contacts than in C12C12 and C16C16. Hence, although displaying larger values for both degree and closeness centrality than C12C1, those values for C16C1 remain far below the ones displayed by C12C12 and C16C16.

The graphs describing the connections between DPPC molecules in the final structures were also computed (Figure 10) and show how the different cations affect the lipid–lipid contacts. It is important to emphasize here that traditional MD tools, like the radial distribution function (Figure S13, between DPPC tails), cannot capture the structural perturbations induced by the cations, while GT can. Since there are two bilayers on each system, two separate networks were obtained for each graph. The cations C4C1, C8C1, C12C1, and C4C4 display essentially the same patterns, with only small shifts of both degree and closeness centrality distributions to slightly smaller values as the size of the alkyl group increases, which agree well with the variations of DPPC-cation and DPPC–DPPC interaction energy discussed before. Overall, those ILs induce relatively small perturbations over the bilayer structure even at high concentrations, which is also reflected in the relatively small changes in the bilayer thickness (Figure 6).

**Figure 10:**
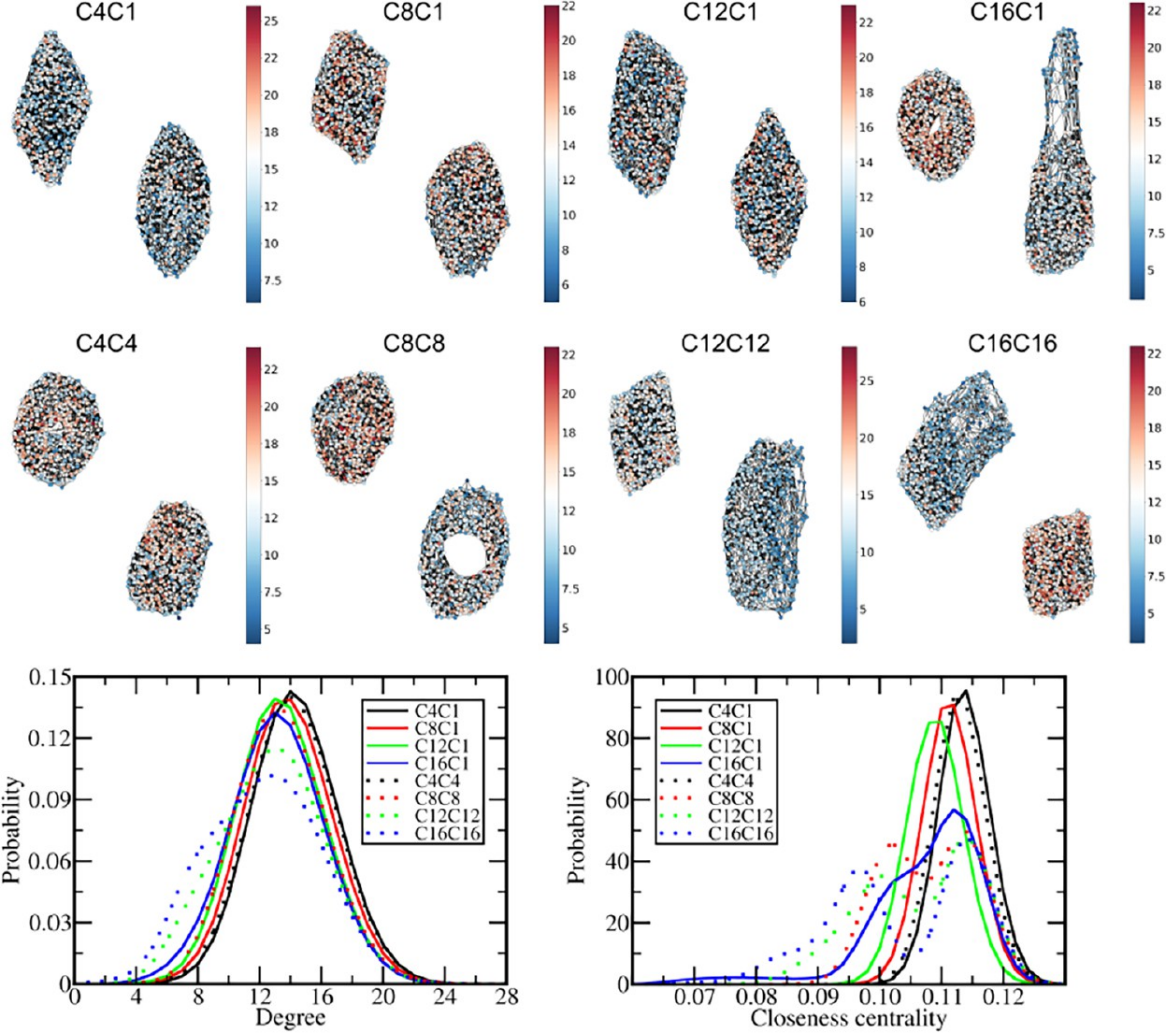
Graph theory description of the DPPC lipid networks. Top: Graphs representing the connection network between DPPC tails in the final structure of each system with 320 ion pairs colored based on the degree values. Bottom: Histograms displaying the distribution of degree and closeness centrality values for the DPPC network along the final 200 ns of the simulations in the systems with 320 ion pairs.

On the other hand, C8C8, C12C12, C16C16, and C16C1 induced deeper changes in the DPPC network. Since those cations penetrate essentially all in a single bilayer, the one without cations or with a few cations remains as a highly connected and compact network, but significant distortions are noticed in the network of the cation-rich bilayer. In C16C1, the lipids that are pulled by the IL semidisk constitute a more spread region of the network, with smaller values for both degree and closeness centrality. However, although many connections between DPPC molecules are broken in this bilayer, no DPPC molecule was completely disconnected from the others. Even being pulled by the IL, those DPPC molecules maintain some contact with each other and with the rest of the bilayer. In C8C8, although a large variation is noticed in the closeness centrality histogram, the distortions remain uniform along the bilayer with the cations. C12C12 and C16C16 networks in the IL-rich bilayer display both a more spread region, corresponding to the lipids around the IL phase, which lose contact with the lipid in the other layer while retaining the lateral contacts with lipids at the same layer, and a more compact region, where only some cations are dispersed.

GT thus enables a rich description of the cation–cation dispersion or segregation in the bilayers as well as of the deformations in the DPPC network due to cation penetration, showing that the lipid network is robust and resists even when large morphology changes are observed. Overall, both degree and closeness centrality capture the same trends on those systems, although the closeness centrality, a nonlocal property, is more sensitive to structural changes, as noticed for phase transitions in surfactant and liquid crystal systems in a previous work.^50^

### 3.4 Anion Penetration

While the previous sections focused on the cation penetration since it is the ion with amphiphilic character, the counterion interaction with the bilayer is also relevant, especially considering the perspective of IL with pharmacologically active ingredients that can penetrate into cellular membranes as an anion associated with an amphiphilic cation that facilitates its penetration.

The anion distribution alongside the coordinate ξ perpendicular to the bilayer with the highest cation concentration in the concentrated systems displays two peaks in the side facing the external solution (negative ξ in Figure 11), while for dilute systems, only the peak close to −2.8 nm is noticed (Figures S5 to S12). The peak at −2.8 nm corresponds to the anions that are adsorbed outside the layer of DPPC phosphate groups, interacting mainly with DPPC ammonium groups, while the peak around −1.8 nm corresponds to anions that penetrate inside the bilayer, interacting mainly with the IL cations. In dilute systems, the cation–anion association is less relevant (radial distribution function in Figure S18), and most anions remain adsorbed outside the phosphate layer. On the other hand, in concentrated systems, most anions penetrate the phosphate layer except in the case of C4C1, for which many cations also remain in aqueous solution. In both cases, an exponential decay in anion concentration is noticed as moving away from the bilayer, as expected from a diffuse electric double layer, although for C16C1, this profile is masked by the formation of the IL semidisk outside the bilayer. For C12C12 and C16C16, a huge anion density is found at the center of the bilayer (ξ = 0 nm) due to the IL phase separation and penetration inside the bilayer as discussed previously.

**Figure 11:**
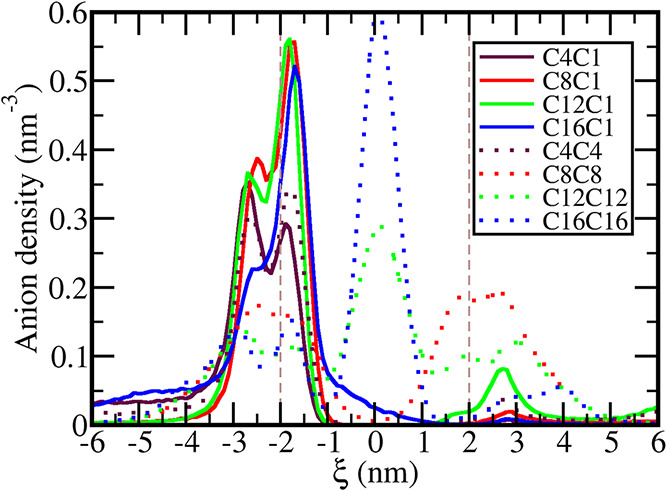
Anion distribution. Density profile for BF_4_^–^ anions in relation to the distance ξ from the bilayer center in the model system with 320 ion pairs. All curves shown correspond to the bilayer with the larger number of cations of the IL incorporated and negative ξ corresponds to the side of the external solution, while positive ξ corresponds to one of the internal solution. Vertical dashed lines indicate the position of the maximum of the DPPC phosphate group on both sides of the bilayer in the system with C8C1 ionic liquid.

The BF_4_^–^ density at positive ξ values corresponds to anions that can pass through the bilayers. In every concentrated system, at least some anions were able to spontaneously reach the internal solution within 1 μs, but the concentration of anions in the internal solution depends strongly on the nature of the cation interaction with the bilayers. Only for C8C8 and C12C12, the anion density becomes similar in the internal and external solutions, indicating that the anion penetration is fast for those ILs, while all the others would demand much longer time scales to reach osmotic equilibrium with the same concentration on both sides of the bilayer. This result was already expected since the cation distributions are also asymmetric (Figure 5). A significant number of anions also passed through the bilayer in C12C1 and C8C1 systems, and, in those cases, we noticed that most of the anions in the internal solution are outside the phosphate layer interacting preferably with DPPC ammonium group due to the lower cation concentration on the side of the bilayer facing the internal solution.

By tracking the amount of anions at each volume defined by the layers of DPPC phosphate groups (Figure 1f) at each recorded frame of the simulation, the anion concentration was computed in the external solution, in the internal solution, and inside the bilayers as a function of time (Figure 12). Similar trends were also noticed for the cation concentration (Figure S24 in the Supporting Information file), except for the fact that the cation concentration in the internal solution is always negligible. For this calculation, the position of the imidazolium group center of geometry was used to define whether the cation is inside the bilayers or in the external or internal solutions. Due to the similarity presented by the two sets, only the concentration of the anion will be discussed here.

**Figure 12:**
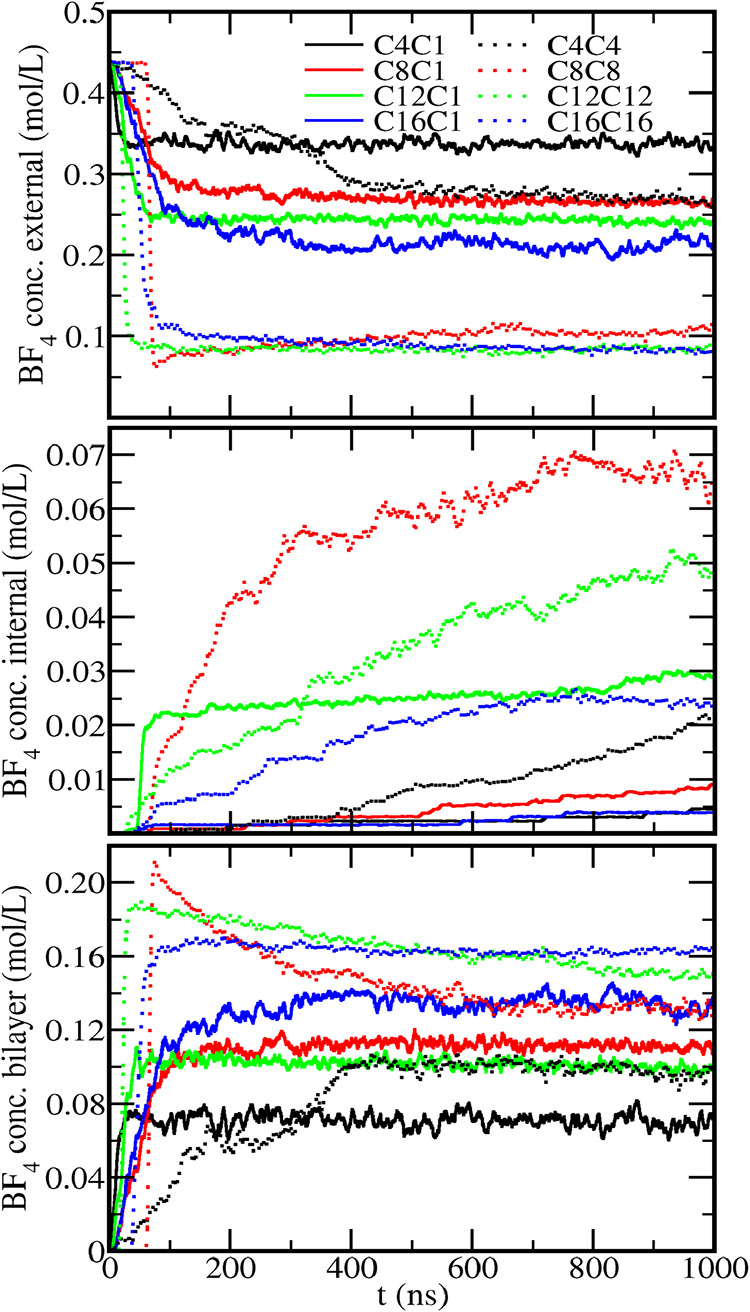
Dynamics of the anion penetration. BF_4_^–^ concentration in external solution (top), internal solution (middle), and inside the lipid bilayers (bottom) along the simulations. A running average was performed at every 12 frames interval to reduce the noise.

All systems started with the same anion concentration in the external solution and no anion inside the bilayer or in the internal solution. For the water-soluble ILs, a progressive decrease is noticed in the anion concentration in the external solution, as they are incorporated in the bilayer and, eventually, in the internal solution individually or carried in small clusters with the cations. For the ILs that presented a phase separation (C16C1, C8C8, C12C12, C16C16), the concentration in the internal solution remained constant for tens of nanoseconds until a sudden drop happens as the whole nanodroplet penetrates inside the bilayer, showing two distinct dynamics of the IL penetration.

After the penetration into the bilayer, the anion needs to cross the hydrophobic center of the bilayer to reach the internal solution, which is slower than the initial incorporation into the bilayer, at least in the case of the BF_4_^–^ anion. This process is reversible: after diffusion to the internal solution, the anion can penetrate the bilayer again, although with small probability as far as the cation density in the internal side remains small, and eventually diffuses back to the external solution.

Hence, two distinct mechanisms were noticed for the anion penetration, which are schematized in Figure 13 together with additional structures for C12C1 and C12C12, focusing on the anion distribution. For water-soluble ILs, the anions diffuse toward the bilayers either individually or adsorbed into micelles formed by the cations, a process that happens in a few nanoseconds. This process is reversible, being observed exchanges between adsorbed and dispersed anions. Due to the relatively high free energy barrier to cross the hydrophobic center of the bilayer, the passage from the external to the internal layer of the membrane is the rate-determining step. Once in the internal side of the bilayer, the exchange of adsorbed anions with the solution is relatively fast. For water-insoluble ILs, on the other hand, a quick phase separation took place in few nanoseconds, and the whole droplet diffuses and penetrates into the bilayer carrying the anions inside. Hence, the initial penetration of the nanodroplet in this case is irreversible and also a relatively fast step. The rate-determining step becomes the diffusion of the anions from the incorporated IL nanodroplet inside the hydrophobic core toward either the internal or external solutions.

**Figure 13:**
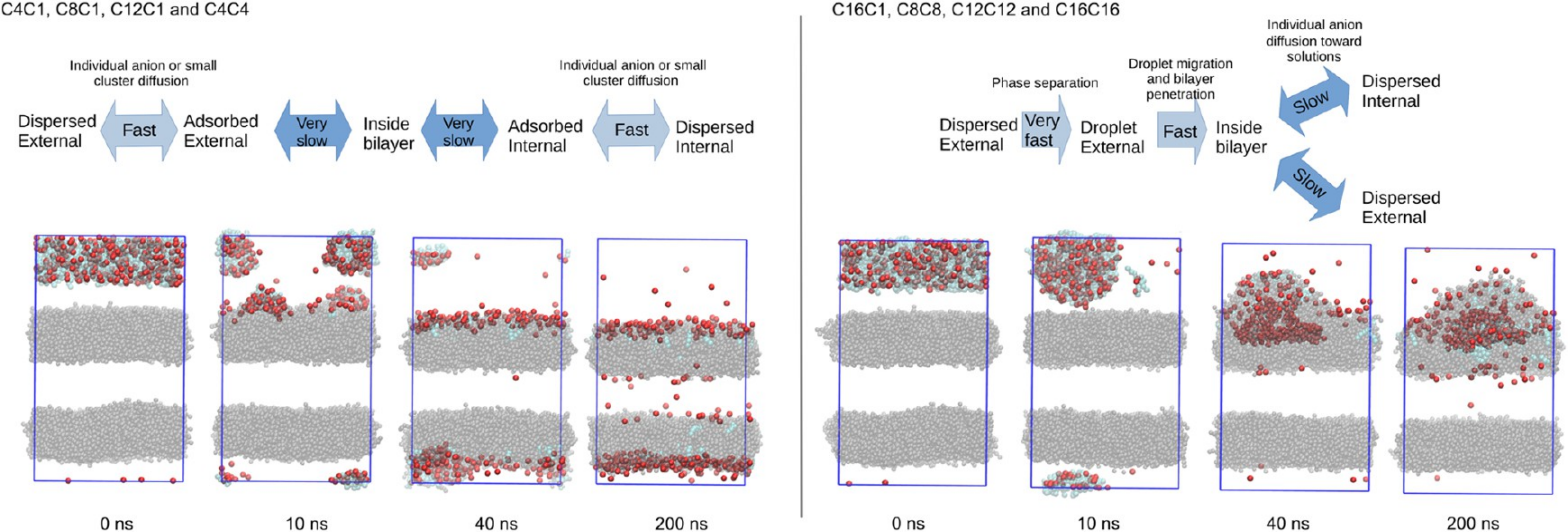
Anion penetration mechanism. Schematic representation of the two proposed mechanisms for IL anion penetration with selected structures along the simulations, with the corresponding times highlighting the anions as solid red spheres while lipid and cations are shown as transparent gray and cyan surfaces, respectively. Left: Mechanism for water-soluble ILs with selected structures for C12C1 shown below. Right: Mechanism for low water solubility ILs with selected structures for C12C12 shown below.

Those results are consistent with the model proposed by Drücker et al. for a different class of imidazolium-based ILs with long-tail substitutions in carbons 4 and 5 of imidazolium ring, which render a parallel orientation between alkyl tails, being more similar to a lipid molecule geometry.^51^ Drücker et al. observed that cations with long alkyl tails (15 carbon atoms) formed vesicles and those vesicles merged with the bilayer, while cations with short tails (7 carbon atoms) migrate either individually or as small micelles toward the lipid bilayer. Due to the geometry of our cations, with an open angle between tails to mimic substitutions at the positions 1 and 3 (nitrogen atoms) of the imidazolium ring, our ILs with two tails are not expected to form vesicles, as observed for their ILs, going to the formation of nanodroplets instead. However, once a nanodroplet or a vesicle is formed, the whole cluster migrates and merges with the bilayer instead of presenting individual cation penetration.

Some caution is necessary regarding this second mechanism. First, it only becomes relevant in concentrated systems; in dilute solutions, all of the ILs studied followed the first mechanism. Also, generating homogeneous solutions for low solubility ILs in those concentrations as in the starting structure of the simulations would not be possible in practical applications either as drug delivery or as an antimicrobial agent. In the best scenario, the low solubility IL would be present as a microemulsion with much larger IL droplets that may lead to different effects. Either way, the water solubility and the possibility of self-assembly in aqueous solutions are shown to be important factors controlling the kinetics of ILs acting as drug delivery.

## 4 Conclusions

The computer simulations performed enable a deep comprehension of the effects of cation alkyl group size and quantity over the penetration of ionic liquids into lipid bilayers and their potential mechanisms to act either as a drug delivery or as an antimicrobial agent. The use of graph theory also proves to be capable of revealing and quantifying structural patterns that are not captured by usual molecular dynamics analysis tools. Besides the expected increase of the thermodynamic tendency to be incorporated into the bilayer as increasing the cation alkyl group size, we noticed that increasing the alkyl group does not affect the activation barrier to bypass the hydrophobic center of the bilayer. Including a second alkyl group, however, leads to a significant reduction and, hence, faster diffusion of the cation between the internal and external sides of the bilayer. Also, the presence of two alkyl groups leads to larger morphology changes and perturbations in the lipid network in comparison to the cations with only one alkyl group. Cations with two long tails lead to a phase separation inside the bilayer depending on the concentration, resulting in a significant increase in the bilayer thickness.

Among the studied ILs, the ones with two alkyl tails lead to a fast penetration of the anions into the internal solution, mainly due to the different mechanism and smaller barrier for cation to traverse the bilayer. Either for the cations with one or two alkyl tails, the faster anion penetration happens for intermediate-sized alkyl groups between 8 and 12 carbon atoms. Small alkyl groups lead to limited cation incorporation into the bilayer and smaller perturbations of the DPPC network, while large alkyl groups lead to strong incorporation of the IL inside the bilayer and a small tendency of the ions to diffuse back to the interface. In both cases, anion transport toward the internal solution becomes inefficient. Hence, cations with intermediate-size chains are more efficient for drug delivery, and despite the fact that cations with two alkyl groups lead to even faster anion penetration, the significant morphology changes they induce into the bilayer would lead to deleterious effects for the cell, and those may be more suitable as antibacterial or sterilization agents.

Further studies are needed to comprehend how the structure of the anion and its interaction with the cation affect transport through the bilayers, as well as consider more realistic biological membrane models, especially to investigate how different lipids and membrane proteins will interact with the ionic liquids and affect both the membrane penetration and morphology changes induced by the ionic liquid. Also, the effects of the bilayer composition on the interaction with ionic liquids need to be further explored. The presence of cholesterol molecules, for instance, can increase the cation penetration and enhance the perturbation effects induced by the ionic liquids, as demonstrated in a recent work,^52^ and IL also affect the transportation of water and small ions through protein channels, as demonstrated by recent atomistic simulations.^27^

## Supplementary Material



## Notes

Additional structures for the potential of mean force calculations, for the systems with 16 ion pairs and for the liquid crystal phase of the C16C1, density profiles for both concentrated and dilute systems, radial distribution function between selected interaction sites, description of graph theory properties with examples in bidimensional lattices, and sampling histograms from the PMF calculations (PDF)

## References

[1] Araújo J. M. M., Florindo C., Pereiro A. B., Vieira N. S. M., Matias A. A., Duarte C. M. M., Rebelo L. P. N., Marrucho I. M. (2014). Cholinium-Based
Ionic Liquids with Pharmaceutically Active Anions. RSC Adv..

[2] Huang W., Wu X., Qi J., Zhu Q., Wu W., Lu Y., Chen Z. (2020). Ionic Liquids: Green
and Tailor-Made Solvents in Drug Delivery. Drug
Discovery Today.

[3] Md
Moshikur R., Chowdhury M. R., Moniruzzaman M., Goto M. (2020). Biocompatible Ionic Liquids and Their Applications in Pharmaceutics. Green Chem..

[4] Sangiorgi S., Albertini B., Bertoni S., Passerini N. (2025). An Overview
on the Role of Ionic Liquids and Deep Eutectic Solvents in Oral Pharmaceuticals. Pharmaceutics.

[5] Krossing I., Slattery J. M., Daguenet C., Dyson P. J., Oleinikova A., Weingärtner H. (2006). Why Are Ionic
Liquids Liquid? A Simple Explanation
Based on Lattice and Solvation Energies. J.
Am. Chem. Soc..

[6] Bernardino K., Zhang Y., Ribeiro M. C., Maginn E. J. (2020). Effect of Alkyl-Group
Flexibility on the Melting Point of Imidazolium-Based Ionic Liquids. J. Chem. Phys..

[7] Philippi F., Welton T. (2021). Targeted Modifications in Ionic Liquids – from
Understanding to Design. Phys. Chem. Chem. Phys..

[8] Lu B., Liu T., Wang H., Wu C., Chen H., Liu Z., Zhang J. (2022). Ionic Liquid Transdermal
Delivery System: Progress, Prospects, and
Challenges. J. Mol. Liq..

[9] Jiang Z., Liu S., Yuan S., Zhang H., Yuan S. (2024). Models of the Three-Component
Bilayer of Stratum Corneum: A Molecular Simulation Study. J. Phys. Chem. B.

[10] Wu H., Deng Z., Zhou B., Qi M., Hong M., Ren G. (2019). Improved Transdermal Permeability
of Ibuprofen by Ionic Liquid Technology:
Correlation between Counterion Structure and the Physicochemical and
Biological Properties. J. Mol. Liq..

[11] Janus E., Ossowicz P., Klebeko J., Nowak A., Duchnik W., Kucharski Ł., Klimowicz A. (2020). Enhancement of Ibuprofen Solubility
and Skin Permeation by Conjugation with L-Valine Alkyl Esters. RSC Adv..

[12] Bani-Jaber A., Hamdan I., Al-Khalidi B. (2007). Sodium Mefenamate
as a Solution for
the Formulation and Dissolution Problems of Mefenamic Acid. Chem. Pharm. Bull. (Tokyo).

[13] Fang Z., Zheng X., Li L., Qi J., Wu W., Lu Y. (2022). Ionic Liquids: Emerging Antimicrobial
Agents. Pharm. Res..

[14] Pałkowski Ł., Karolak M., Skrzypczak A., Wojcieszak M., Walkiewicz F., Podemski J., Jaroch K., Bojko B., Materna K., Krysiński J. (2022). Antimicrobial
and Cytotoxic Activity
of Novel Imidazolium-Based Ionic Liquids. Molecules.

[15] Mao X., Auer D. L., Buchalla W., Hiller K.-A., Maisch T., Hellwig E., Al-Ahmad A., Cieplik F. (2020). Cetylpyridinium Chloride:
Mechanism of Action, Antimicrobial Efficacy in Biofilms, and Potential
Risks of Resistance. Antimicrob. Agents Chemother..

[16] Soumet C., Fourreau E., Legrandois P., Maris P. (2012). Resistance to Phenicol
Compounds Following Adaptation to Quaternary Ammonium Compounds in *Escherichia coli*. Vet. Microbiol..

[17] Grenier M. C., Davis R. W., Wilson-Henjum K. L., LaDow J. E., Black J. W., Caran K. L., Seifert K., Minbiole K. P. C. (2012). The Antibacterial
Activity of 4,4′-Bipyridinium Amphiphiles with Conventional,
Bicephalic and Gemini Architectures. Bioorg.
Med. Chem. Lett..

[18] Ator L. E., Jennings M. C., McGettigan A. R., Paul J. J., Wuest W. M., Minbiole K. P. C. (2014). Beyond Paraquats:
Dialkyl 3,3′- and 3,4′-Bipyridinium
Amphiphiles as Antibacterial Agents. Bioorg.
Med. Chem. Lett..

[19] Amata S., Calà C., Rizzo C., Pibiri I., Pizzo M., Buscemi S., Palumbo Piccionello A. (2024). Synthesis and Antibacterial Activity
of Mono- and Bi-Cationic Pyridinium 1,2,4-Oxadiazoles and Triazoles. Int. J. Mol. Sci..

[20] Hassanpour M., Torabi S. M., Afshar D., Kowsari M. H., Meratan A. A., Nikfarjam N. (2024). Tracing the
Antibacterial Performance of Bis-Imidazolium-Based
Ionic Liquid Derivatives. ACS Appl. Bio Mater..

[21] Sharma V. K., Gupta J., Mitra J. B., Srinivasan H., Sakai V. G., Ghosh S. K., Mitra S. (2024). The Physics of Antimicrobial
Activity of Ionic Liquids. J. Phys. Chem. Lett..

[22] Kirchner B., Hollóczki O., Canongia Lopes J. N., Pádua A. A. H. (2015). Multiresolution
Calculation of Ionic Liquids. WIREs Comput.
Mol. Sci..

[23] Yoo B., Shah J. K., Zhu Y., Maginn E. J. (2014). Amphiphilic Interactions
of Ionic Liquids with Lipid Biomembranes: A Molecular Simulation Study. Soft Matter.

[24] Forero
Doria O., Castro R., Gutierrez M., Gonzalez Valenzuela D., Santos L., Ramirez D., Guzman L. (2018). Novel Alkylimidazolium
Ionic Liquids as an Antibacterial Alternative to Pathogens of the
Skin and Soft Tissue Infections. Molecules.

[25] Lim G. S., Jaenicke S., Klähn M. (2015). How the Spontaneous Insertion of
Amphiphilic Imidazolium-Based Cations Changes Biological Membranes:
A Molecular Simulation Study. Phys. Chem. Chem.
Phys..

[26] Ganjali
Koli M., Azizi K. (2016). The Partition and Transport Behavior of Cytotoxic Ionic
Liquids (ILs) through the DPPC Bilayer: Insights from Molecular Dynamics
Simulation. Mol. Membr. Biol..

[27] Liu J., Ren J., Li S., He H., Wang Y. (2024). Protein Interface Regulating
the Inserting Process of Imidazole Ionic Liquids into the Cell Membrane. J. Phys. Chem. B.

[28] Liu J., Wang Y., Gao B., Zhang K., Li H., Ren J., Huo F., Zhao B., Zhang L., Zhang S., He H. (2024). Ionic Liquid
Gating Induces Anomalous Permeation through Membrane
Channel Proteins. J. Am. Chem. Soc..

[29] Domańska U., Bogel-Łukasik E., Bogel-Łukasik R. (2003). 1-Octanol/Water Partition Coefficients
of 1Alkyl-3-Methylimidazolium Chloride. Chem.
- Eur. J..

[30] Vila J., Fernández-Castro B., Rilo E., Carrete J., Domínguez-Pérez M., Rodríguez J. R., García M., Varela L. M., Cabeza O. (2012). Liquid–Solid–Liquid
Phase Transition Hysteresis Loops in the Ionic Conductivity of Ten
Imidazolium-Based Ionic Liquids. Fluid Phase
Equilib..

[31] Vazquez-Salazar L. I., Selle M., de Vries A. H., J Marrink S. T., Souza P. C. (2020). Martini Coarse-Grained Models of Imidazolium-Based
Ionic Liquids: From Nanostructural Organization to Liquid–Liquid
Extraction. Green Chem..

[32] Souza P. C. T., Alessandri R., Barnoud J., Thallmair S., Faustino I., Grünewald F., Patmanidis I., Abdizadeh H., Bruininks B. M. H., Wassenaar T. A., Kroon P. C., Melcr J., Nieto V., Corradi V., Khan H. M., Domański J., Javanainen M., Martinez-Seara H., Reuter N., Best R. B., Vattulainen I., Monticelli L., Periole X., Tieleman D. P., de Vries A. H., Marrink S. J. (2021). Martini 3: A General Purpose Force Field for Coarse-Grained
Molecular Dynamics. Nat. Methods.

[33] Bernardino K. (2024). How Domain
Segregation in Ionic Liquids Stabilizes Nanoparticles and Establishes
Long-Range Ordering—A Computational Study. ACS Nano.

[34] Silva L. O. X., Bernardino K. (2025). Domain Segregation in Ionic Liquids Induces Long-Range
Oscillatory Forces between Nanoparticles and Surfaces. ACS Nanosci. Au.

[35] Martínez L., Andrade R., Birgin E. G., Martínez J. M. (2009). PACKMOL:
A package for building initial configurations for molecular dynamics
simulations. J. Comput. Chem..

[36] Zhu F., Hummer G. (2012). Convergence and Error
Estimation in Free Energy Calculations
Using the Weighted Histogram Analysis Method. J. Comput. Chem..

[37] Hub J. S., de Groot B. L., van der Spoel D. (2010). G_wham—A Free Weighted Histogram
Analysis Implementation Including Robust Error and Autocorrelation
Estimates. J. Chem. Theory Comput..

[38] Bernardino K., de Moura A. F. (2013). Aggregation
Thermodynamics of Sodium Octanoate Micelles
Studied by Means of Molecular Dynamics Simulations. J. Phys. Chem. B.

[39] Berendsen H. J. C., van der Spoel D., van Drunen R. (1995). GROMACS: A
Message-Passing Parallel Molecular Dynamics Implementation. Comput. Phys. Commun..

[40] Abraham M. J., Murtola T., Schulz R., Páll S., Smith J. C., Hess B., Lindahl E. (2015). GROMACS: High
Performance
Molecular Simulations through Multi-Level Parallelism from Laptops
to Supercomputers. SoftwareX.

[41] Bussi G., Donadio D., Parrinello M. (2007). Canonical
Sampling through Velocity
Rescaling. J. Chem. Phys..

[42] Berendsen H. J. C., Postma J. P. M., van
Gunsteren W. F., DiNola A., Haak J. R. (1984). Molecular
Dynamics with Coupling to an External Bath. J. Chem. Phys..

[43] Essmann U., Perera L., Berkowitz M. L., Darden T., Lee H., Pedersen L. G. (1995). A Smooth Particle
Mesh Ewald Method. J. Chem. Phys..

[44] Humphrey W., Dalke A., Schulten K. (1996). VMD: Visual
Molecular Dynamics. J. Mol. Graph..

[45] Santos D. E. S., Pontes F. J. S., Lins R. D., Coutinho K., Soares T. A. (2020). SuAVE:
A Tool for Analyzing Curvature-Dependent Properties in Chemical Interfaces. J. Chem. Inf. Model..

[46] Freeman L. C. (1978). Centrality
in Social Networks Conceptual Clarification. Soc. Netw..

[47] Hagberg, A. A. ; Schult, D. A. ; Swart, P. J. Exploring Network Structure, Dynamics, and Function Using NetworkX Pasadena, CA, 2008; pp 11–15 10.25080/TCWV9851.

[48] Israelachvili, J. N. Intermolecular and Surface Forces, 3rd ed.; Academic Press: Burlington, MA, 2011.

[49] Kong X., Qin S., Lu D., Liu Z. (2014). Surface Tension Effects on the Phase
Transition of a DPPC Bilayer with and without Protein: A Molecular
Dynamics Simulation. Phys. Chem. Chem. Phys..

[50] Yang R., Bernardino K., Xiao X., Gomes W. R., Mattoso D. A., Kotov N. A., Bogdan P., de Moura A. F. (2024). Graph Theoretical
Description of Phase Transitions in Complex Multiscale Phases with
Supramolecular Assemblies. Adv. Sci..

[51] Drücker P., Rühling A., Grill D., Wang D., Draeger A., Gerke V., Glorius F., Galla H.-J. (2017). Imidazolium Salts
Mimicking the Structure of Natural Lipids Exploit Remarkable Properties
Forming Lamellar Phases and Giant Vesicles. Langmuir.

[52] Gupta J., Sharma V. K., Hitaishi P., Jha A. K., Mitra J. B., Srinivasan H., Kumar S., Kumar A., Ghosh S. K., Mitra S. (2025). Cholesterol Modulates Ionic Liquid–Lipid
Membrane Interactions. Langmuir.

