# Supplementary material for: Ionic Liquids as Antibacterial and Drug Delivery Agents: How Cationic Amphiphilic Structure Controls Morphology Changes in Lipid Bilayers and Penetration Mechanism
Source: J Phys Chem B. 2026 Mar 2;130(10):2782–97. doi: 10.1021/acs.jpcb.5c07740 (PMC12990117; doi:10.1021/acs.jpcb.5c07740)
Supplement: Supplementary file 1 [file jp5c07740_si_001.pdf]

Supporting Information for

Ionic liquids as anti-bacterial and drug delivery agents: How cationic amphiphilic structure controls morphology changes in lipid bilayers and penetration mechanism

*Ludmila Baldan do Rosario,[1] Leticia Rafaella Dias,[1] Andrea Paravani da Costa,[1] Asdrubal Lozada-Blanco,[2†] Kalil Bernardino[1]**

* kalilb@ufscar.br, † alozada@usp.br

1. Laboratório de Química Computacional, Chemistry Department, Universidade Federal de São Carlos, Rod. Washington Luiz S/n, São Carlos, Brazil. Zip code 13565-905

2. Chemistry Department, FFCLRP, University of São Paulo, Av. Bandeirantes 3900, Ribeirão Preto, SP, Brazil. Zip code 14040-901

Contents:

1. Additional structures S2
2. Density profiles S6
3. Radial distribution function between hydrophobic sites S14
4. Radial distribution function between charged sites S16
5. Graph-based analysis of interaction networks S18
6. Sampling histograms of the pmf calculations S21
7. Dynamics of cation penetration into lipid bilayers S23

1. Additional structures

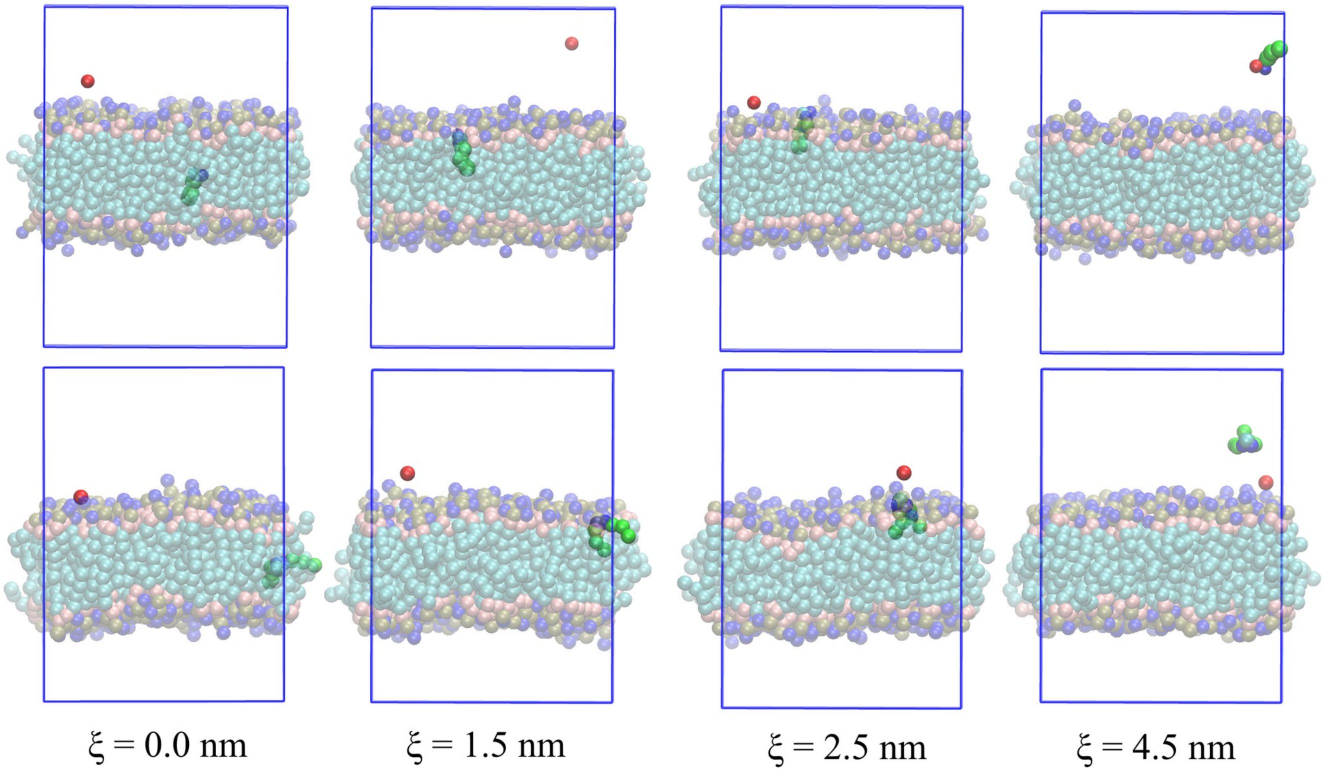

Figure S1 - Selected structures in the PMF calculation for the C12C1 (top) and C12C12 (bottom) ionic liquids at different positions along the reaction coordinate ξ defined as the distance between the DPPC lipid bilayer center and the imidazolium ring of the cation. DPPC molecules are displayed as translucid van der Waals sphere while the ions are displayed as opaque spheres. Water was hidden for better visualization.

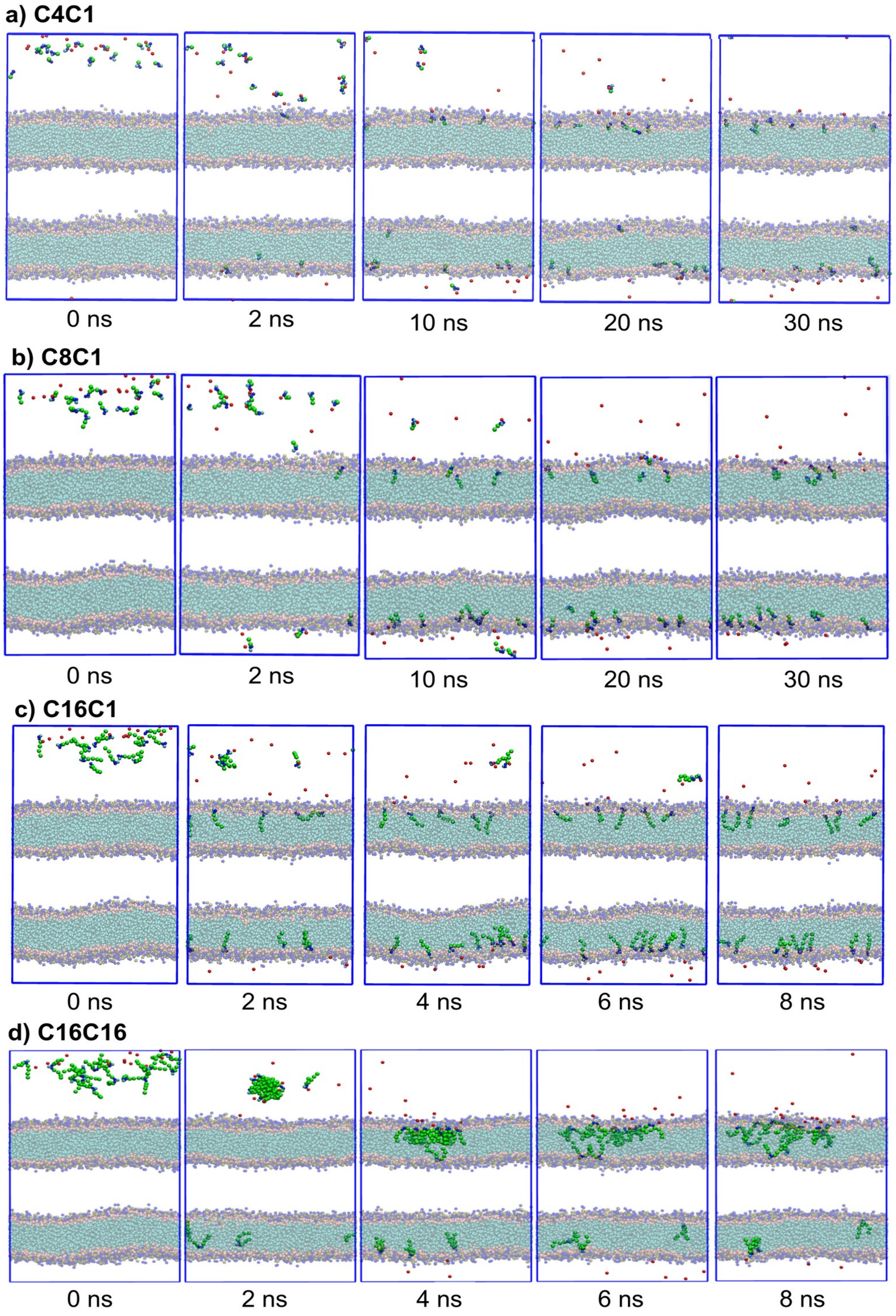

Figure S2 – Selected structures alongside the relaxation of the systems with 16 ion pairs of the ILs **a)** C4C1, **b)** C8C1, **c)** C16C1 and **d)** C16C16. Water was hidden for better visualization.

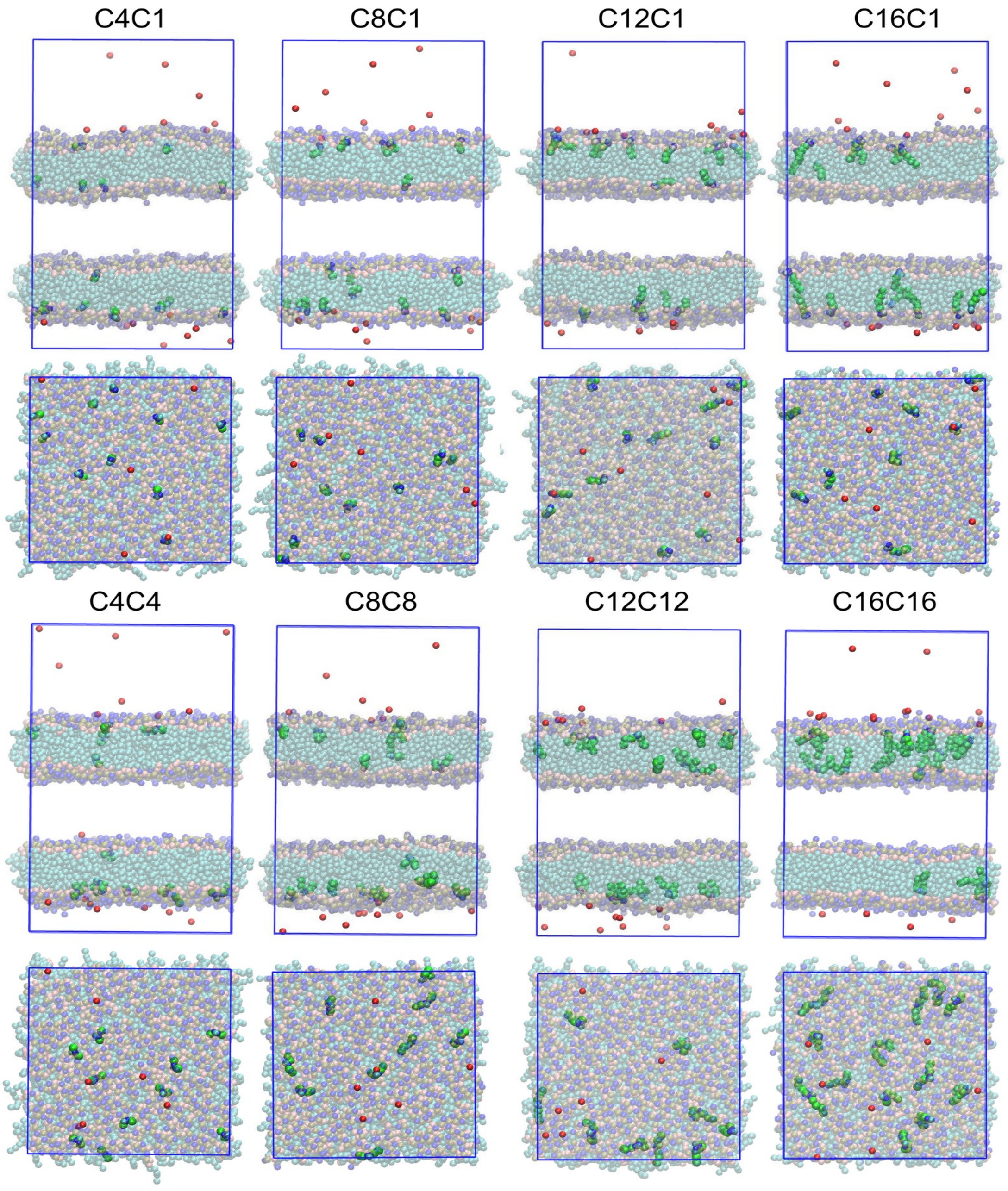

Figure S3 – Lateral and top view from the final structures from molecular dynamics simulations of each ionic liquid interacting with the DPPC bilayer in the models with 16 ion pairs. DPPC lipids are displayed as transparent van der Waals spheres while cations and anions from the ILs are displayed as opaque spheres. Water was hidden for better visualization. In the top views, only the bilayer with the highest cation density is shown with the cations and anions that are in contact with at least one DPPC molecule from the bilayer.

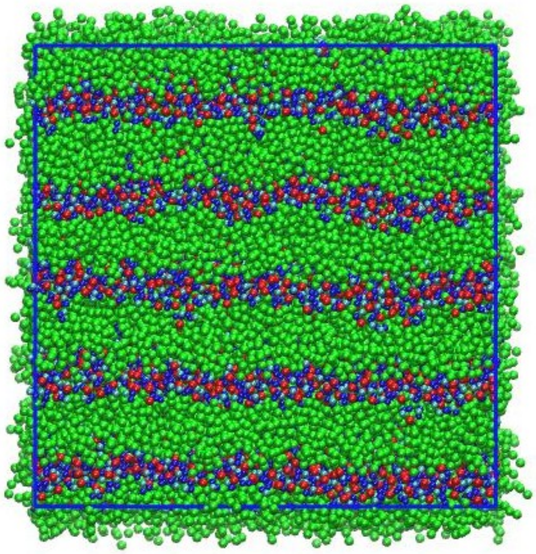

Figure S4 – Smectic liquid crystal phase observed in a pure 1-hexadecyl-3-methylimidazolium tetrafluoroborate (C16C1) liquid 200 ns simulation with 9500 ion pairs and T=330 K. All other simulation conditions were the same employed in the bilayer simulations. Anions are displayed in red, cation alkyl group in green and cation imidazolium sites in blue and cyan.

2. Density profiles

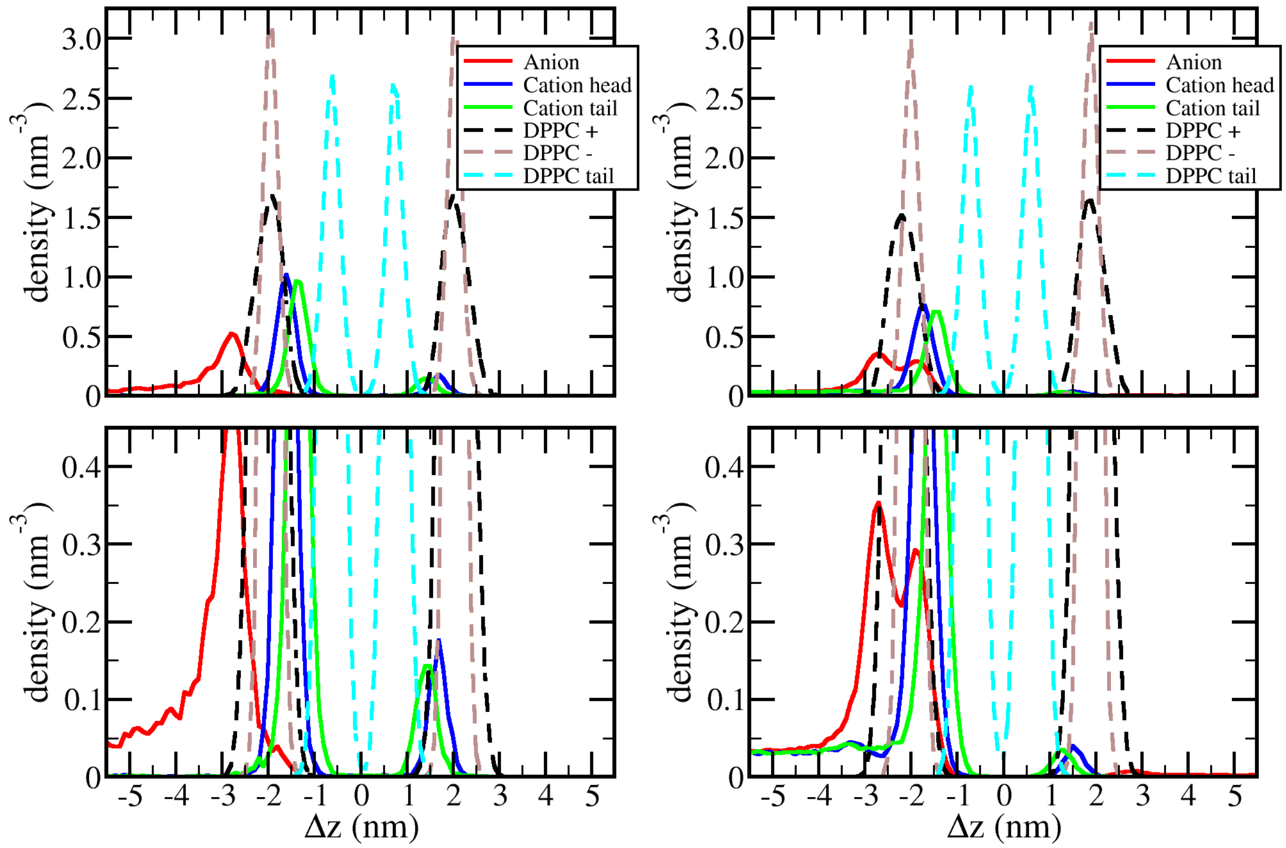

Figure S5 – Density profiles in relation to the distance Δz from the center of the corresponding groups in relation to the center of the bilayer with the highest cation density for the systems with 16 (left) and 320 ion pairs (right) of the IL C4C1, with negative Δz corresponding to the external solution and positive Δz to the internal solution. Bottom panels zooms over small densities and the cation and anion density profiles in the system with 16 ion pairs were multiplied by 20 for better visualization.

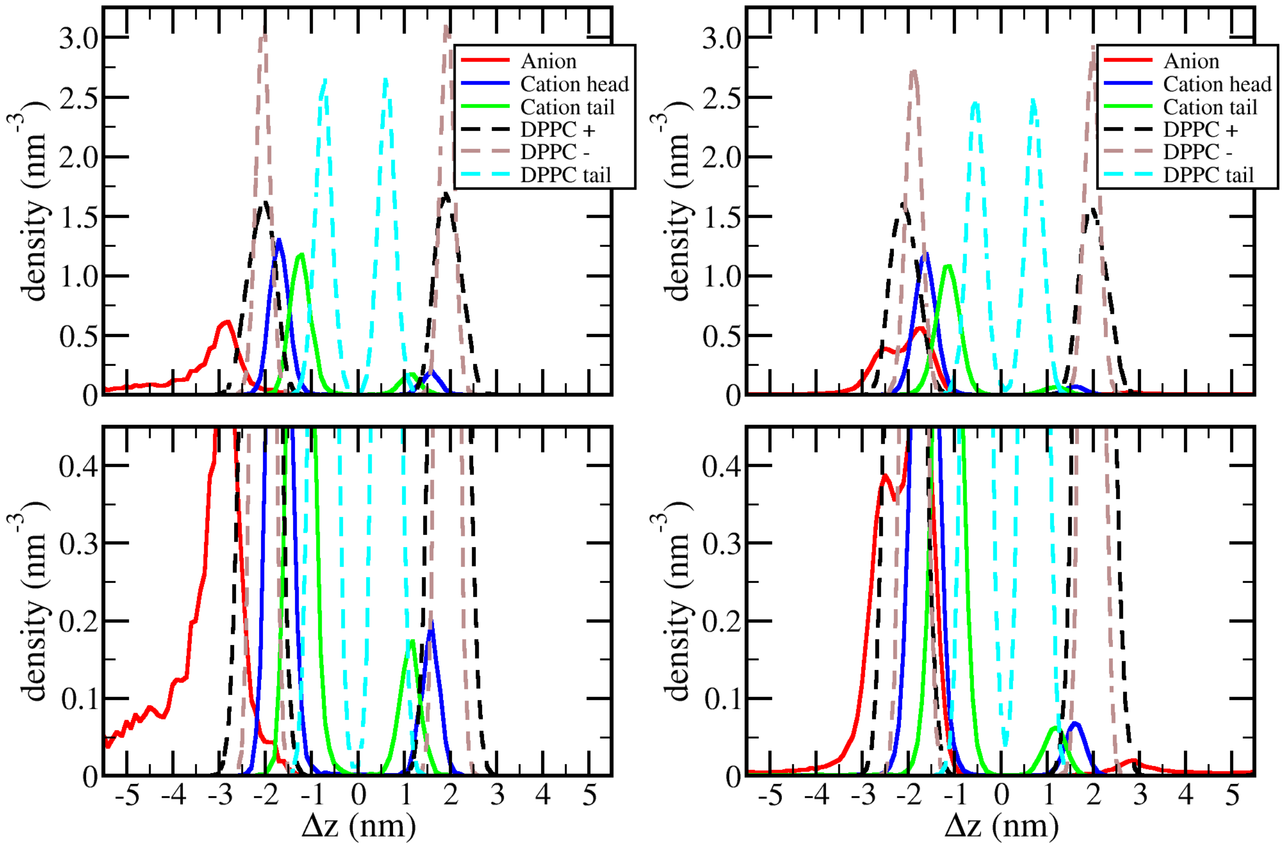

Figure S6 – Density profiles in relation to the distance Δz from the center of the corresponding groups in relation to the center of the bilayer with the highest cation density for the systems with 16 (left) and 320 ion pairs (right) of the IL C8C1, with negative Δz corresponding to the external solution and positive Δz to the internal solution. Bottom panels zooms over small densities and the cation and anion density profiles in the system with 16 ion pairs were multiplied by 20 for better visualization.

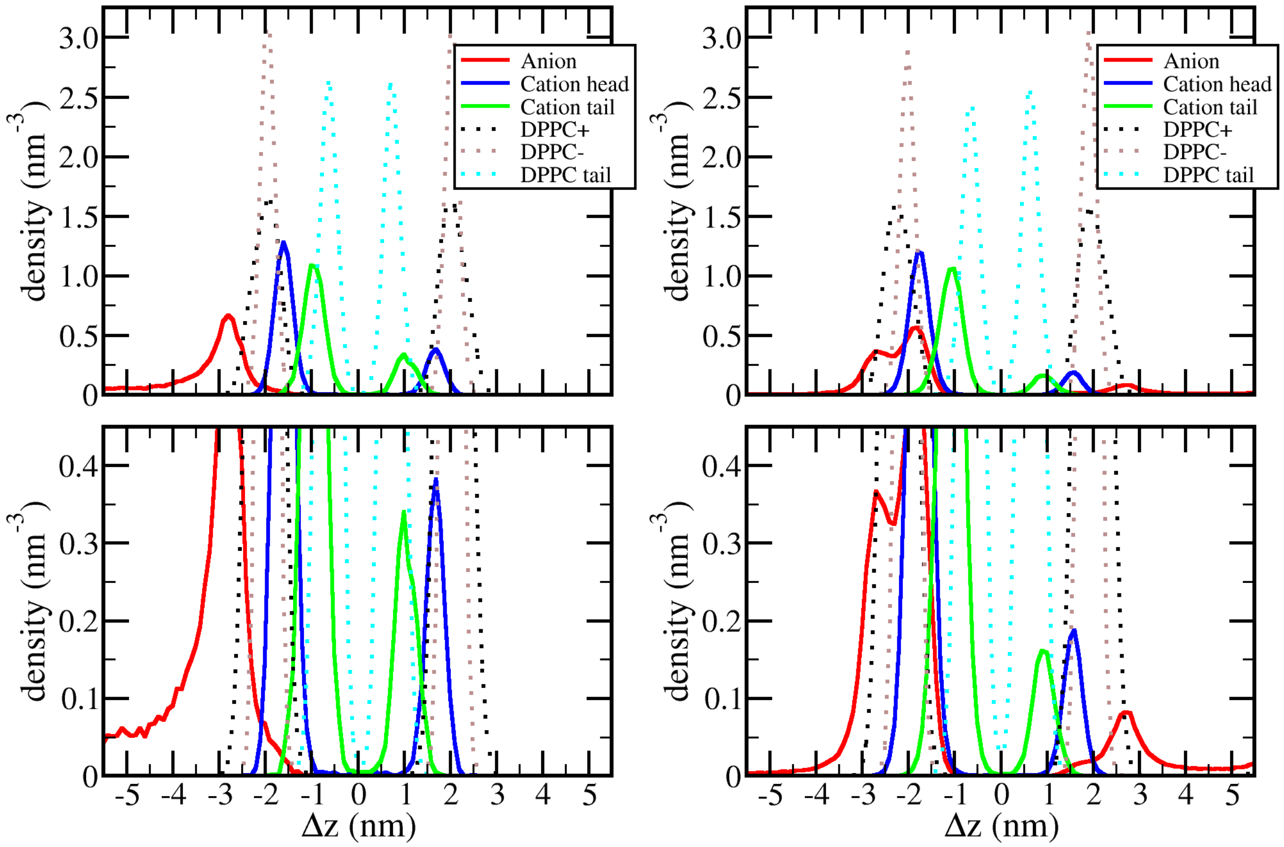

Figure S7 – Density profiles in relation to the distance Δz from the center of the corresponding groups in relation to the center of the bilayer with the highest cation density for the systems with 16 (left) and 320 ion pairs (right) of the IL C12C1, with negative Δz corresponding to the external solution and positive Δz to the internal solution. Bottom panels zooms over small densities and the cation and anion density profiles in the system with 16 ion pairs were multiplied by 20 for better visualization.

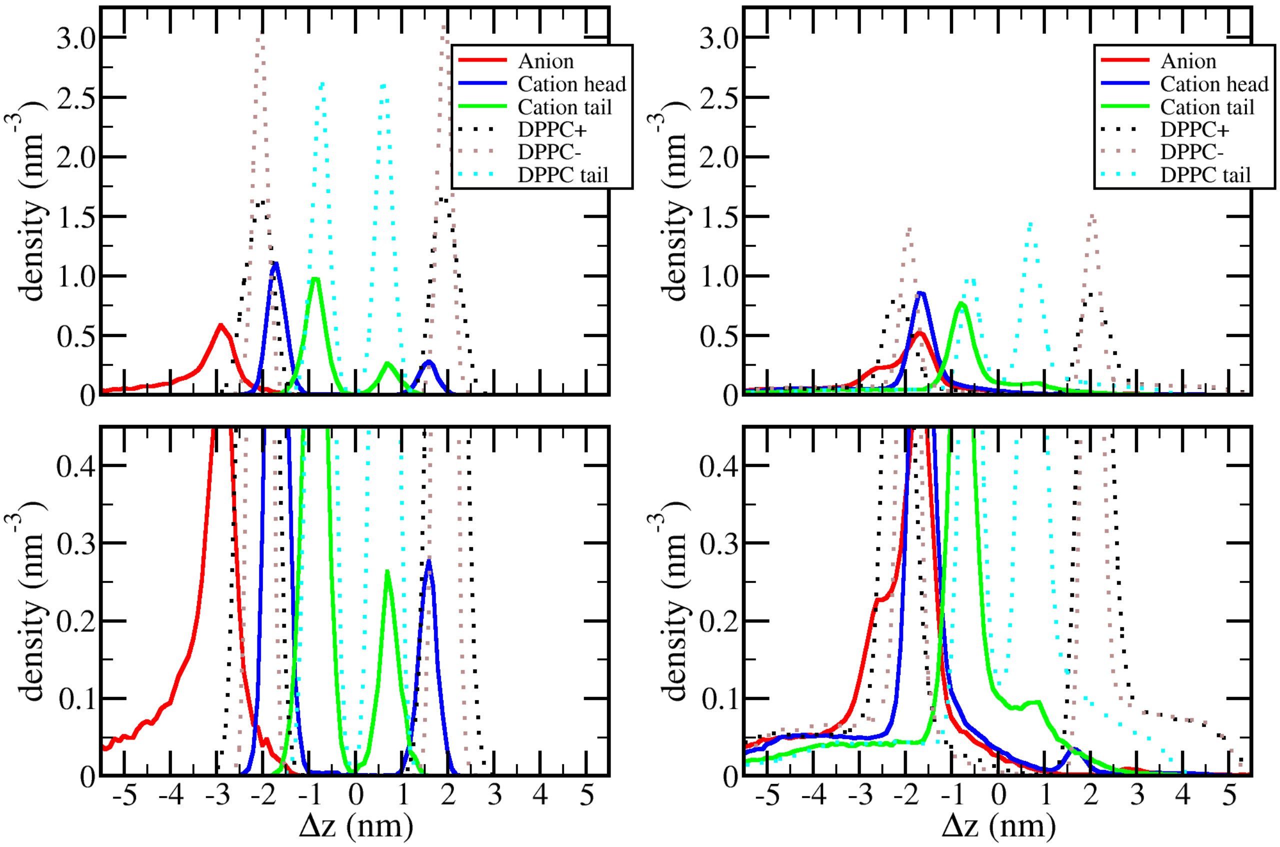

Figure S8 – Density profiles in relation to the distance Δz from the center of the corresponding groups in relation to the center of the bilayer with the highest cation density for the systems with 16 (left) and 320 ion pairs (right) of the IL C16C1, with negative Δz corresponding to the external solution and positive Δz to the internal solution. Bottom panels zooms over small densities and the cation and anion density profiles in the system with 16 ion pairs were multiplied by 20 for better visualization.

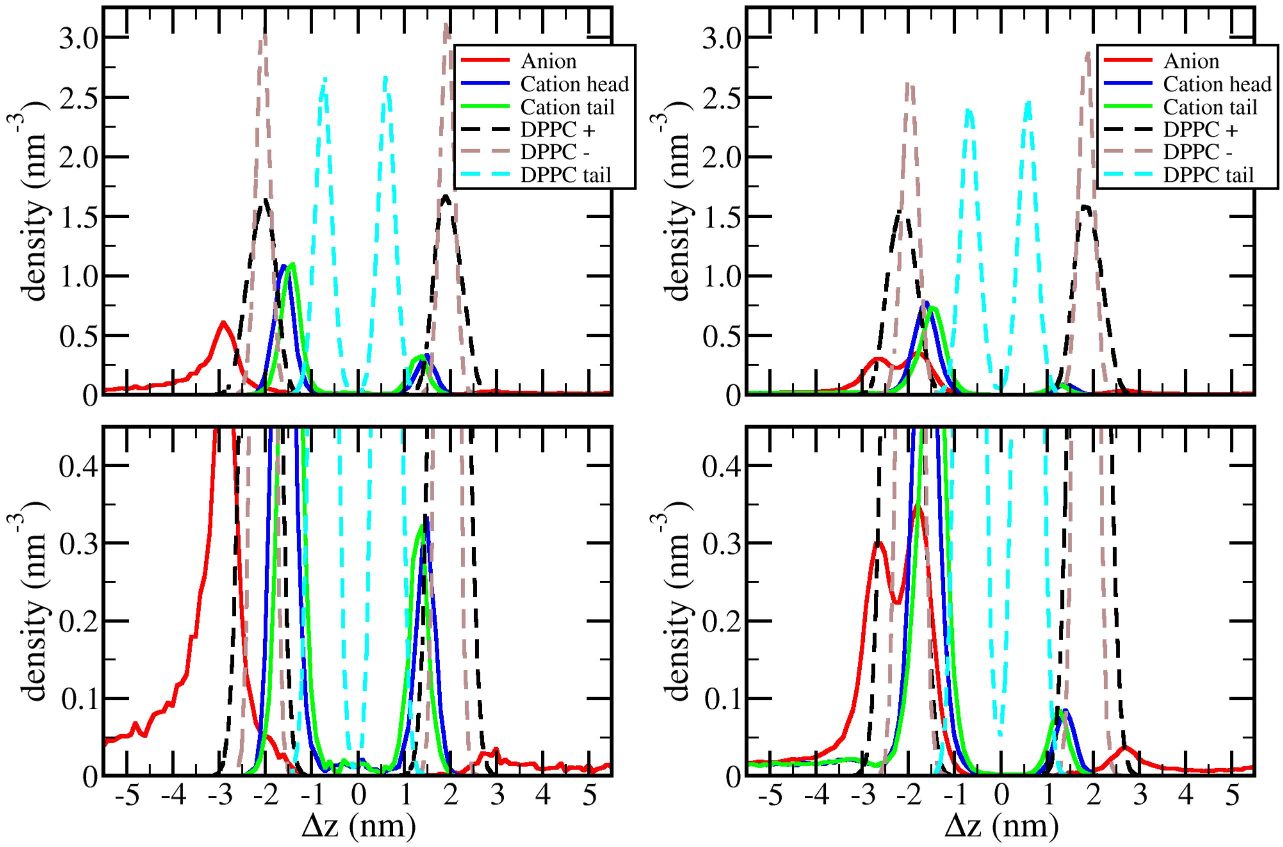

Figure S9 – Density profiles in relation to the distance Δz from the center of the corresponding groups in relation to the center of the bilayer with the highest cation density for the systems with 16 (left) and 320 ion pairs (right) of the IL C4C4, with negative Δz corresponding to the external solution and positive Δz to the internal solution. Bottom panels zooms over small densities and the cation and anion density profiles in the system with 16 ion pairs were multiplied by 20 for better visualization.

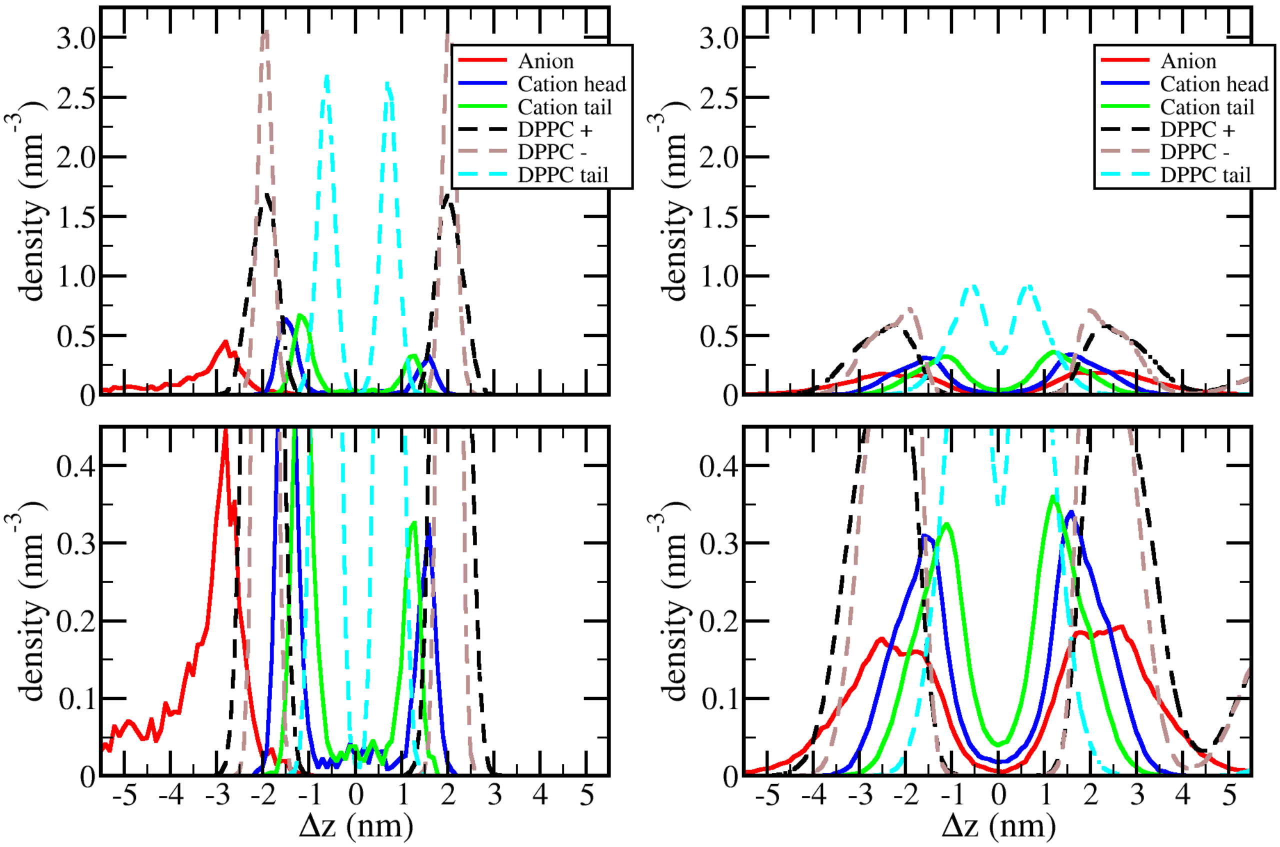

Figure S10 – Density profiles in relation to the distance Δz from the center of the corresponding groups in relation to the center of the bilayer with the highest cation density for the systems with 16 (left) and 320 ion pairs (right) of the IL C8C8, with negative Δz corresponding to the external solution and positive Δz to the internal solution. Bottom panels zooms over small densities and the cation and anion density profiles in the system with 16 ion pairs were multiplied by 20 for better visualization.

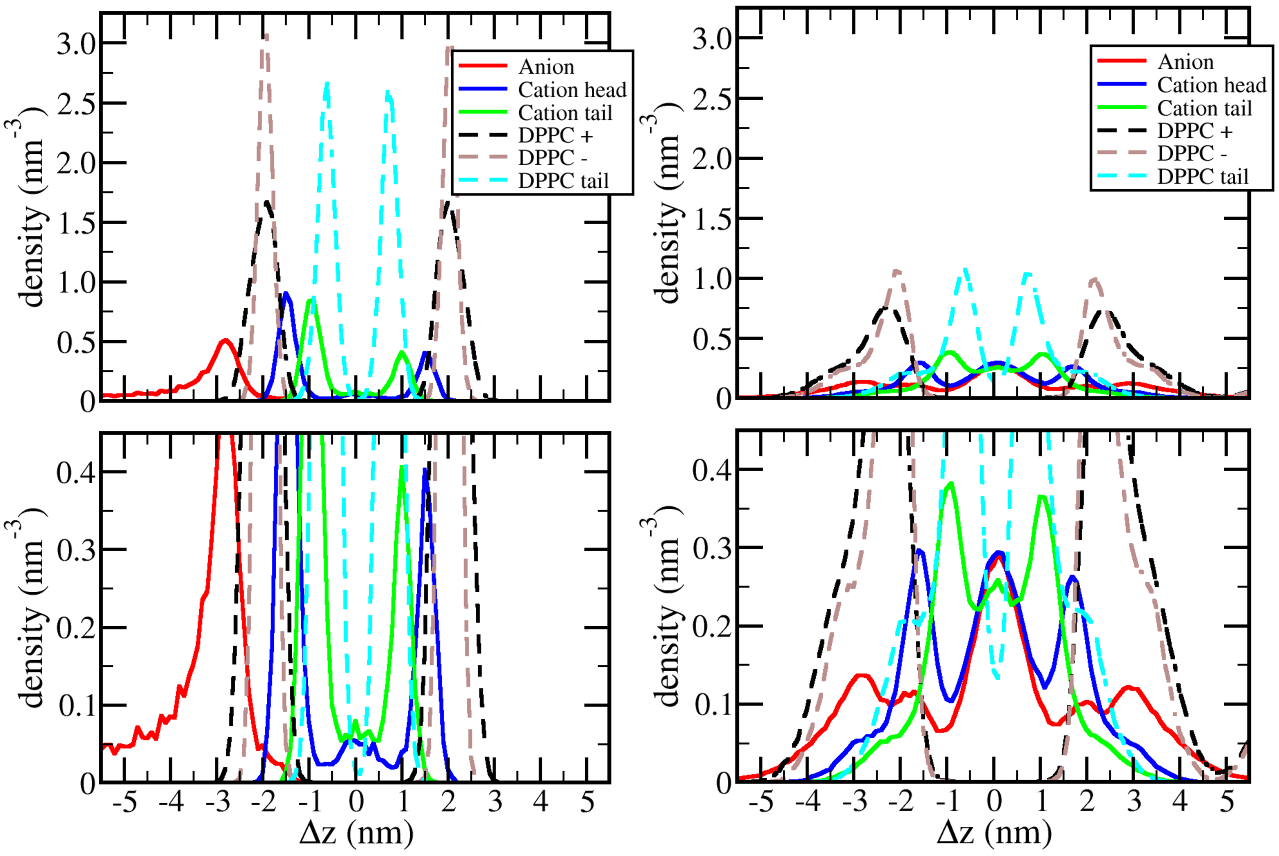

Figure S11 – Density profiles in relation to the distance Δz from the center of the corresponding groups in relation to the center of the bilayer with the highest cation density for the systems with 16 (left) and 320 ion pairs (right) of the IL C12C12, with negative Δz corresponding to the external solution and positive Δz to the internal solution. Bottom panels zooms over small densities and the cation and anion density profiles in the system with 16 ion pairs were multiplied by 20 for better visualization.

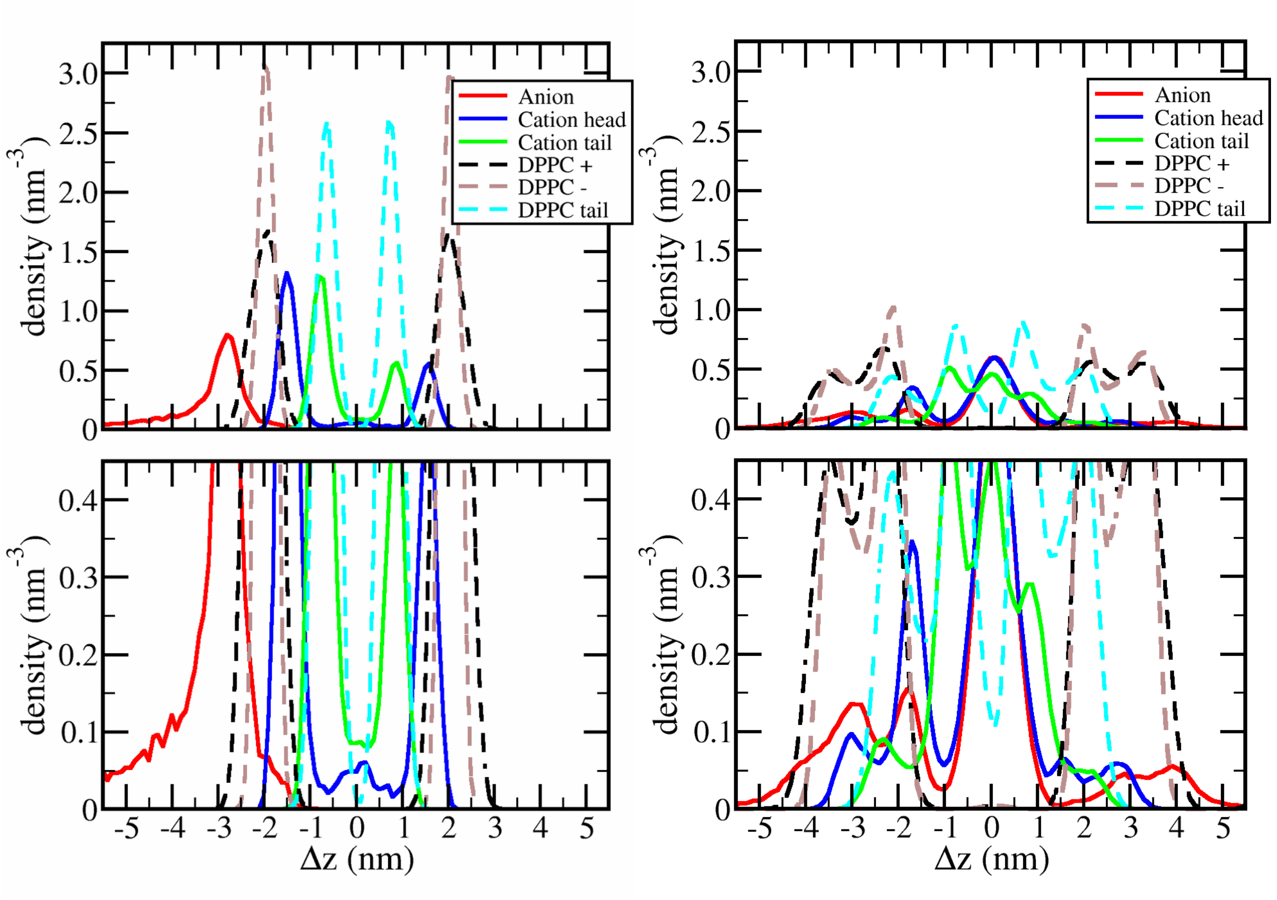

Figure S12 – Density profiles in relation to the distance Δz from the center of the corresponding groups in relation to the center of the bilayer with the highest cation density for the systems with 16 (left) and 320 ion pairs (right) of the IL C16C16, with negative Δz corresponding to the external solution and positive Δz to the internal solution. Bottom panels zooms over small densities and the cation and anion density profiles in the system with 16 ion pairs were multiplied by 20 for better visualization.

3. Radial distribution function between hydrophobic sites

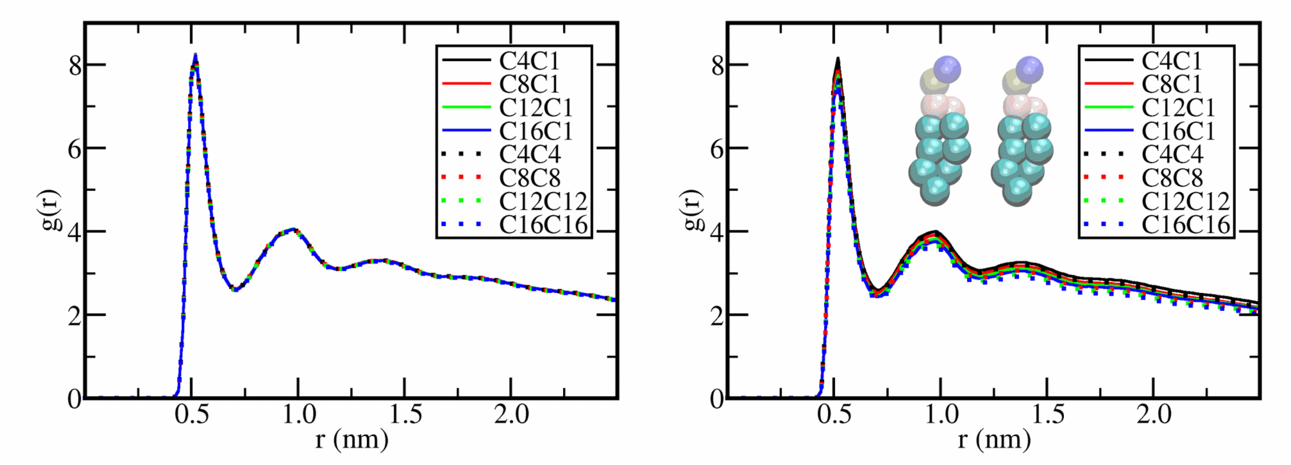

Figure S13 – Radial distribution function between DPPC tail sites (highlighted in the structures displayed) for systems with 16 (left) and 320 (right) ion pairs.

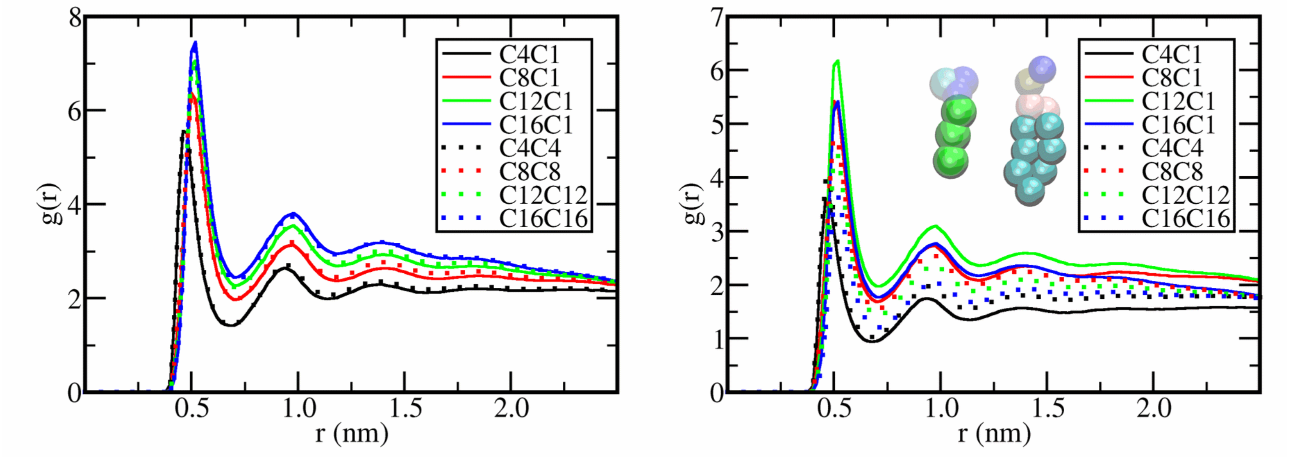

Figure S14 – Radial distribution function between cation and DPPC tail sites (highlighted in the structures displayed using C12C1 as example for the cation) for systems with 16 (left) and 320 (right) ion pairs.

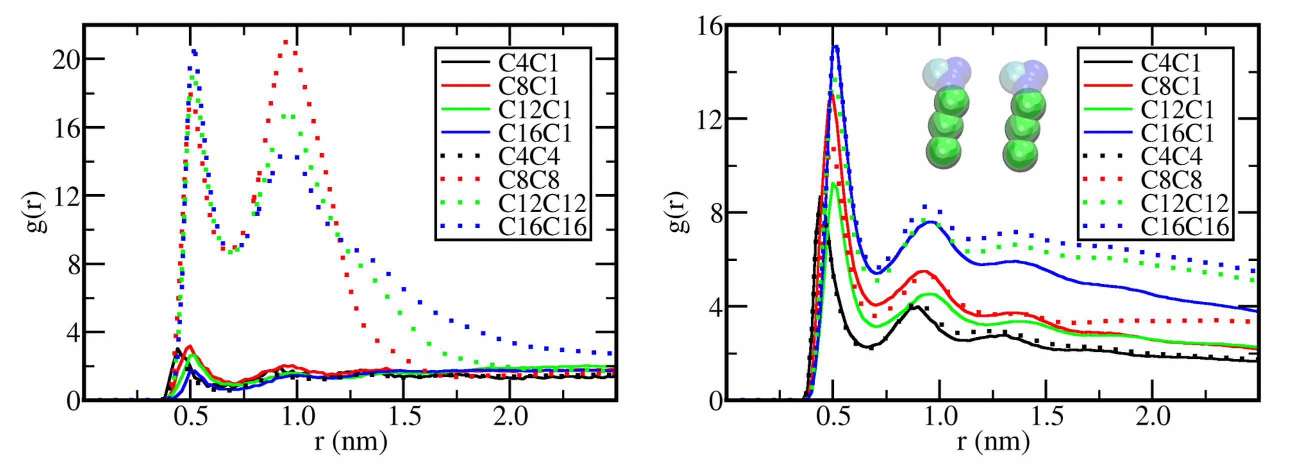

Figure S15 – Radial distribution function between cation tail sites (highlighted in the structures displayed using C12C1 as example for the cation) for systems with 16 (left) and 320 (right) ion pairs.

4. Radial distribution function between charged sites

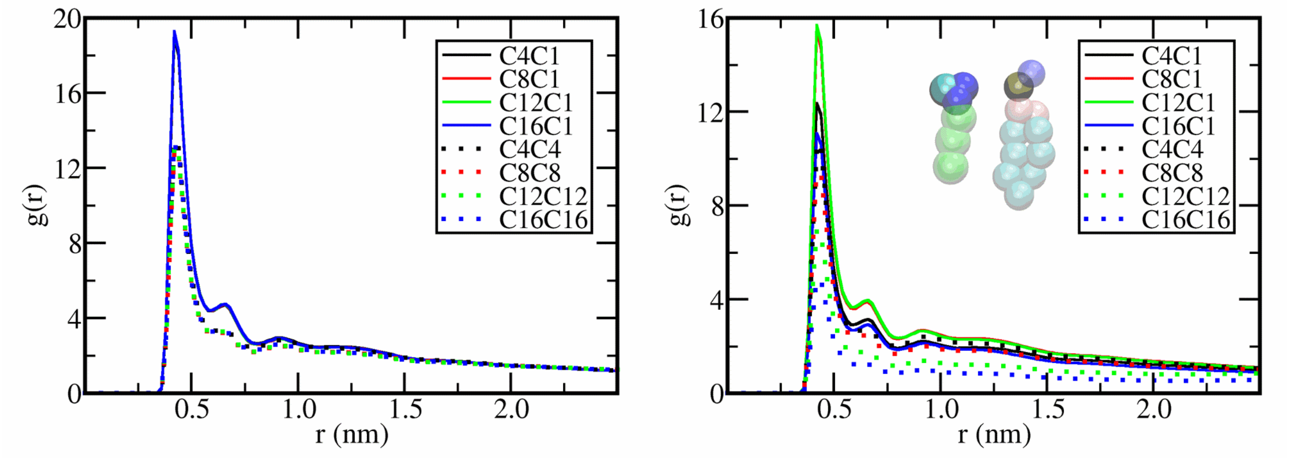

Figure S16 – Radial distribution function between cation imidazolium and DPPC phosphate sites (highlighted in the structures displayed using C12C1 as example for the cation) for systems with 16 (left) and 320 (right) ion pairs.

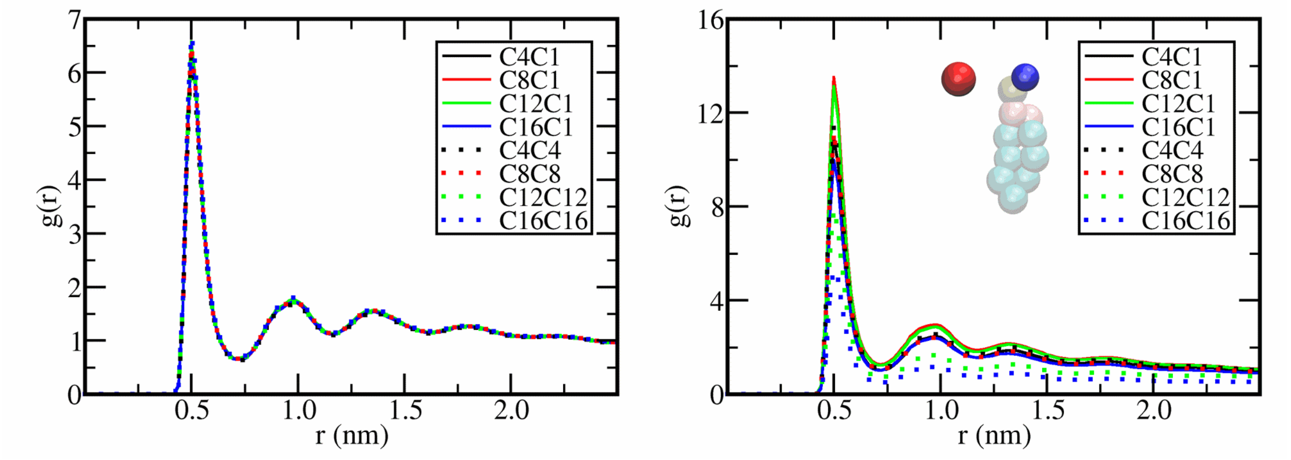

Figure S17 – Radial distribution function between tetrafluoroborate anion and DPPC ammonium sites (highlighted in the structures displayed) for systems with 16 (left) and 320 (right) ion pairs.

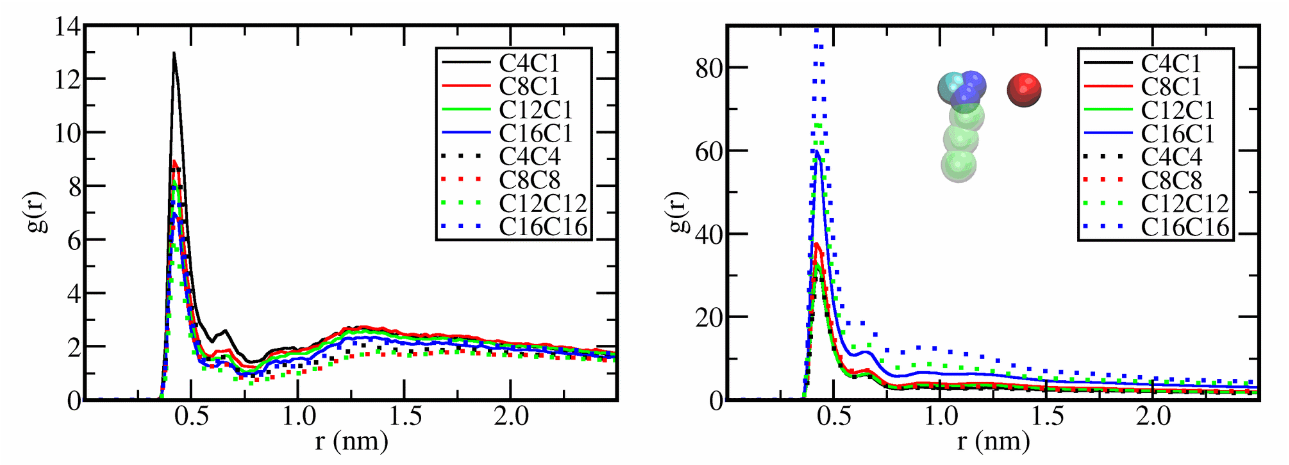

Figure S18 – Radial distribution function between cation imidazolium sites and tetrafluoroborate anion (highlighted in the structures displayed using C12C1 as example for the cation) for systems with 16 (left) and 320 (right) ion pairs.

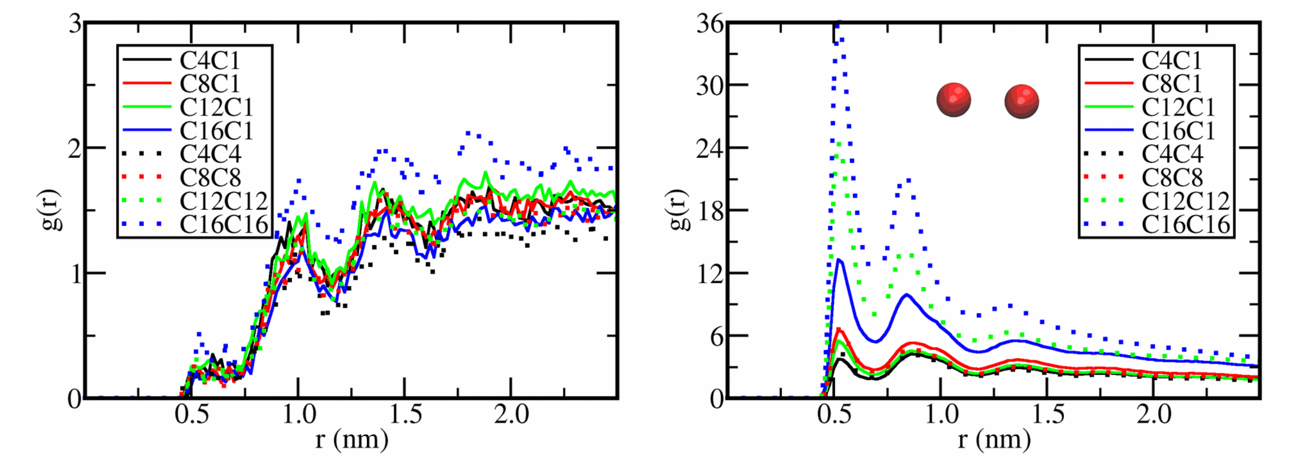

Figure S19 – Radial distribution function between tetrafluoroborate anions for systems with 16 (left) and 320 (right) ion pairs.

5. Graph-based analysis of interaction networks

Networks were built from the molecular dynamics simulation results by defining nodes and linking them based on observed interactions. After these interaction networks were generated, centrality measures were calculated to quantify the relative importance of each node. Degree centrality was used to capture local connectivity by counting the number of direct connections, while closeness centrality assessed global accessibility as the inverse of the average shortest-path distance to all other nodes.

To illustrate how the centrality measures are calculated and interpreted, we provide their definitions along with simple examples in two-dimensional lattices. We consider regular rectangular lattices with 25 sites and variations produced by the removal of one or two sites. These examples help demonstrate how different centrality measures capture various aspects of node importance depending on the structure of the network.

As described by Freeman[34] in the context of structural centrality measures in social networks, degree and closeness centrality are commonly defined for undirected graphs as

$$C_D = \frac{deg(v)}{n-1}$$

and

$$C_C = \frac{n-1}{\sum d(i,j)}$$

respectively, where $deg(v)$ is the number of edges incident on a node or vertex i, $d(i,j)$ is the distance between vertices i and j, and n is the total number of vertices. Notice, however, that in the analyses presented on the manuscript the normalization factor of n – 1 was not used in the calculation of the degree, hence the degree values presented are simply the number of other nodes (ions or molecules) in contact.

The rectangular lattice consists of 25 nodes arranged in a regular grid, with each node connected to its nearest neighbors. This lattice offers a straightforward case of homogeneous connectivity. With periodic boundary conditions (PBC), this lattice can be embedded on a rectangular

torus, preserving its orthogonal geometry and every node has the same values for both degre and closness centrality (Figure S20).

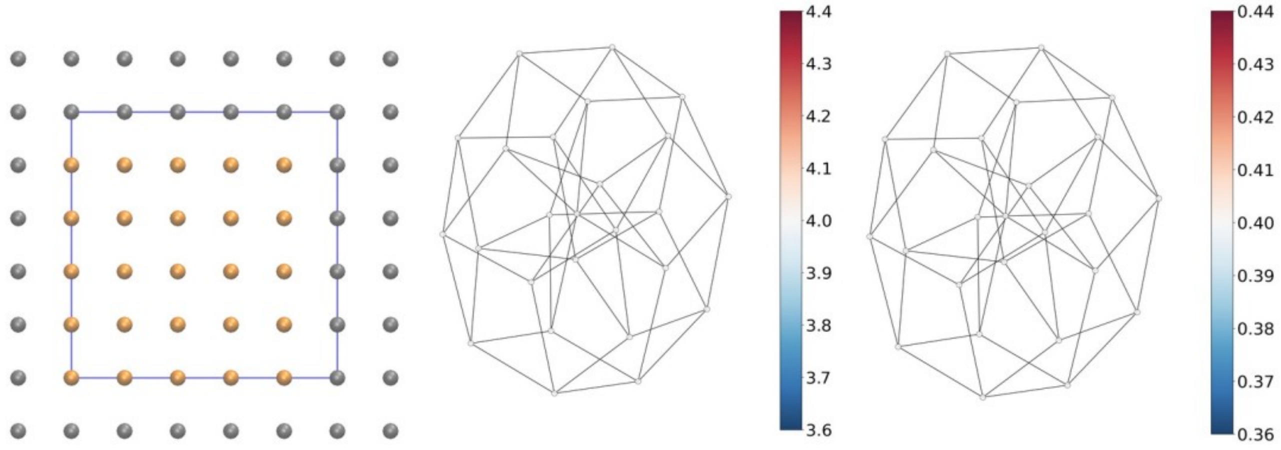

Figure S20 – Left: Regular rectangular bidimensional lattice with the sites inside the box colored in orange and some sites of the periodic replicas displayed in gray. Center: Graph representation colored based on the degree. Right: Graph representation colored based in closeness centrality.

Variations of the rectangular lattice produced by removing one or two sites presents a more heterogeneous topology (Figure S21). The degree of the nodes neighbor to the deleted ones decrease from 4 to 3. Closeness centrality values also decreases since the shortest paths between some pairs of the remaining nodes are not present anymore, thus increasing some d(i,j) values. While the degree affects only the immediate neighborhood of the defect, closeness centrality changes happens for every site, thus, closeness centrality consists in a long-range property while the degree is a local / short-range property.

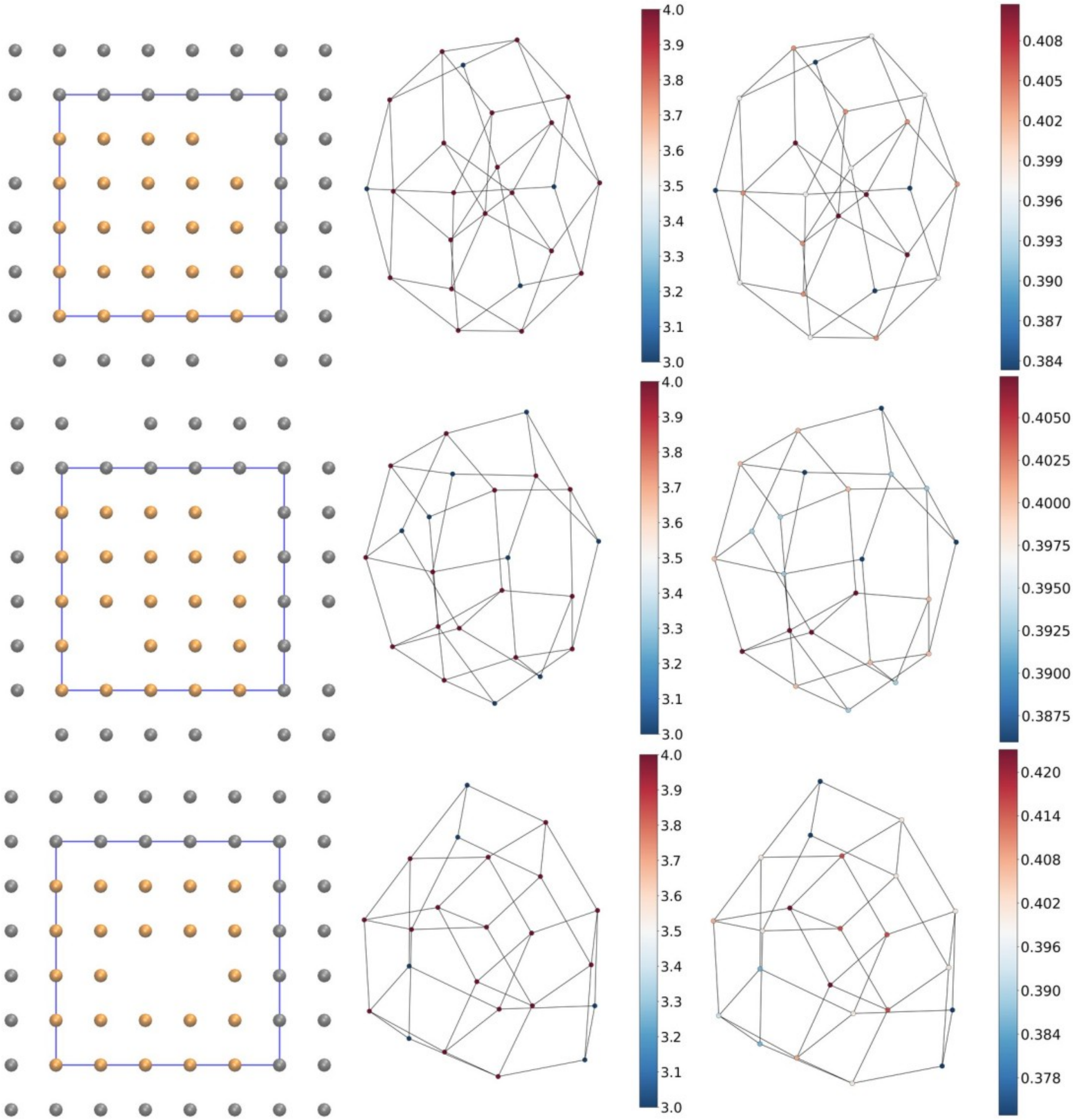

Figure S21 – Left: Rectangular bidimensional lattice with defects with the sites inside the box colored in orange and some sites of the periodic replicas displayed in gray. From top to bottom: One deleted site, two distant sites deleted, two neighbor sited deleted. Center: Graph representation colored based on the degree. Right: Graph representation colored based in closeness centrality.

6. Sampling histograms of the pmf calculations

Sampling histograms displaying the positions of the imidazolium ring center of mass in each window of the umbrella sampling calculations (Figures S22 and S23). The good superposition between consecutive distributions along the reaction coordinate shows that the force constant and the number of sampling window used was appropriate.

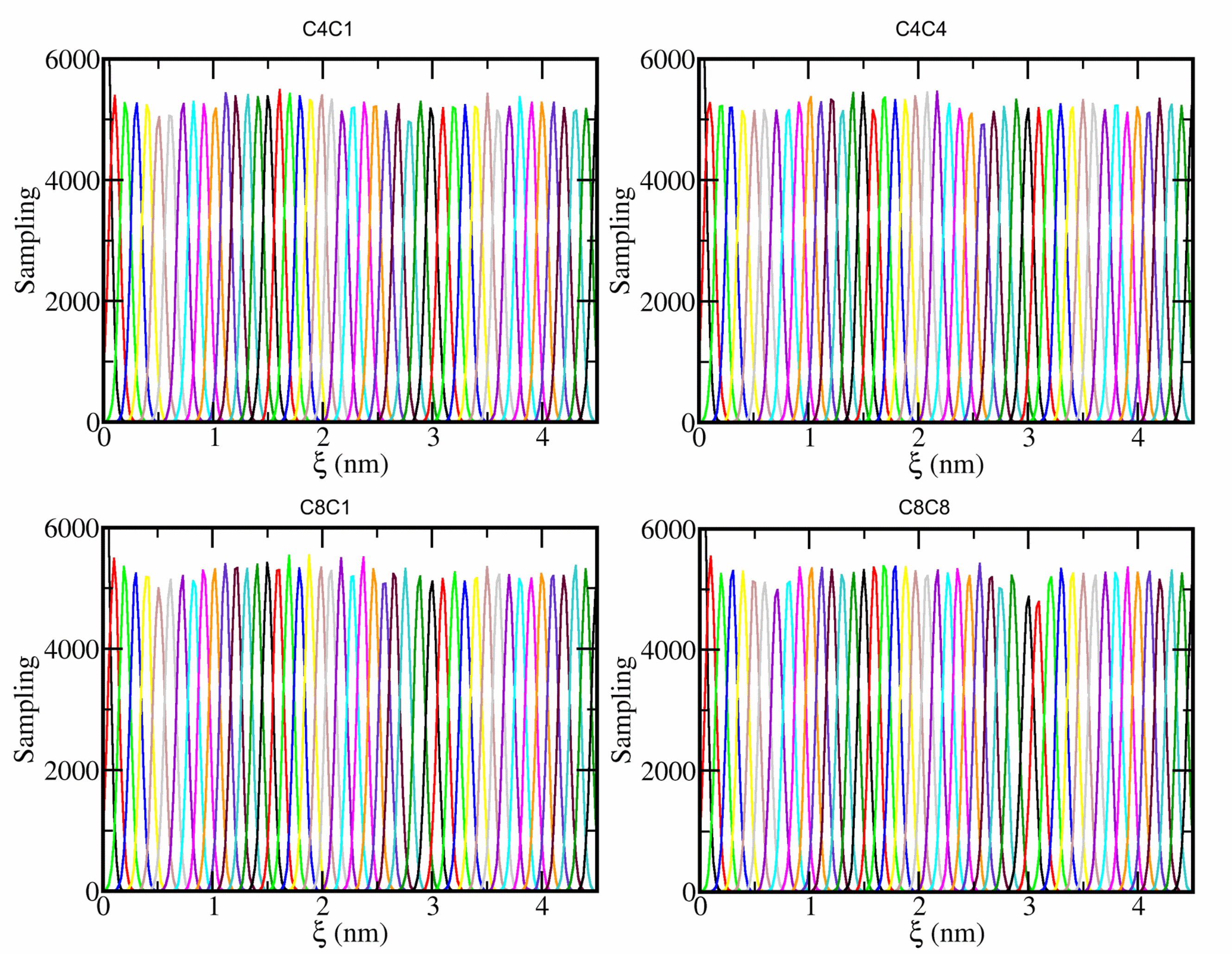

Figure S22 – Sampling along the reaction coordinate ξ for each umbrella sampling window for the ILs C4C1, C4C4, C8C1 and C8C8.

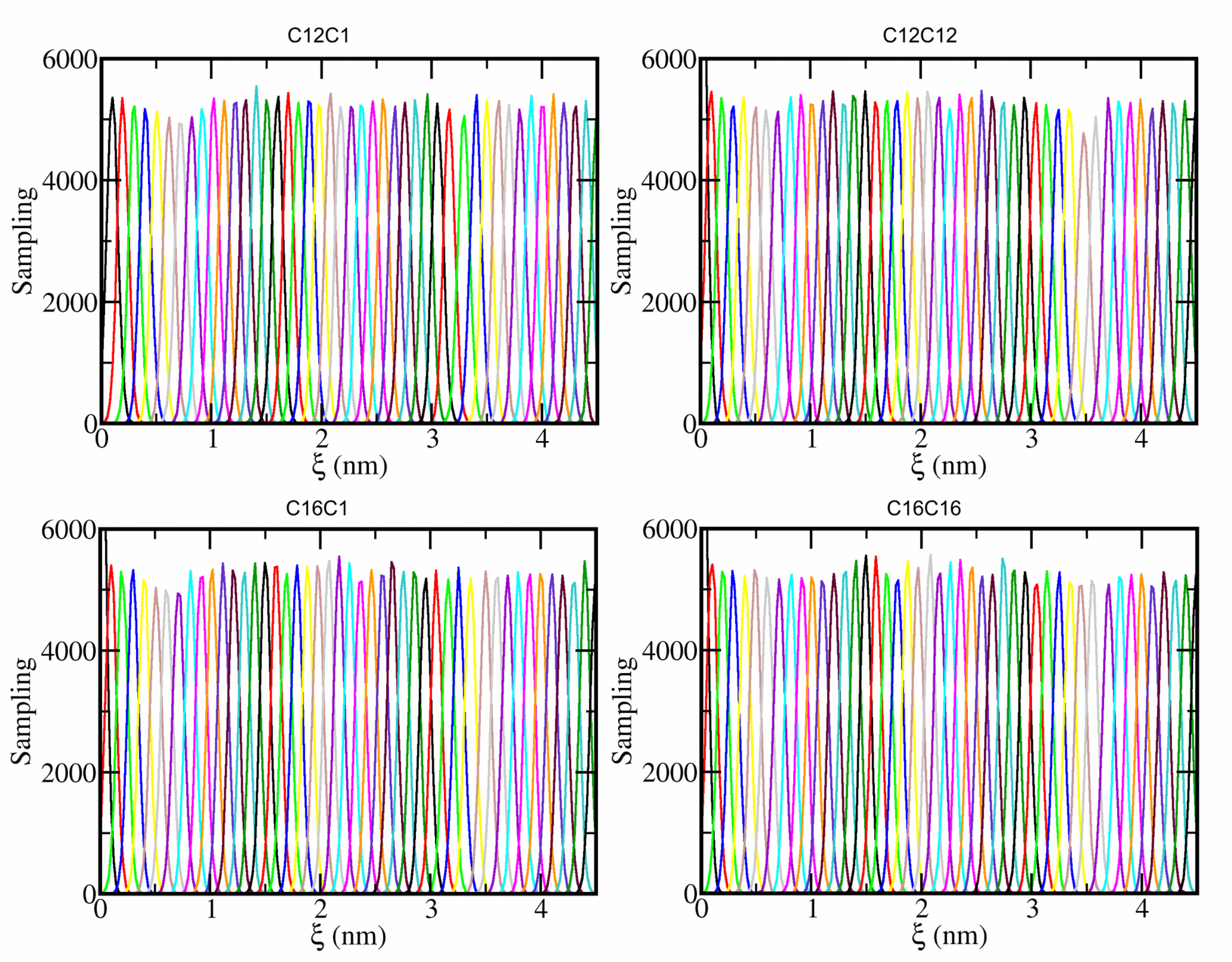

Figure S23 – Sampling along the reaction coordinate ξ for each umbrella sampling window for the ILs C12C1, C12C12, C16C1 and C16C16.

7. Dynamics of cation penetration into lipid bilayers

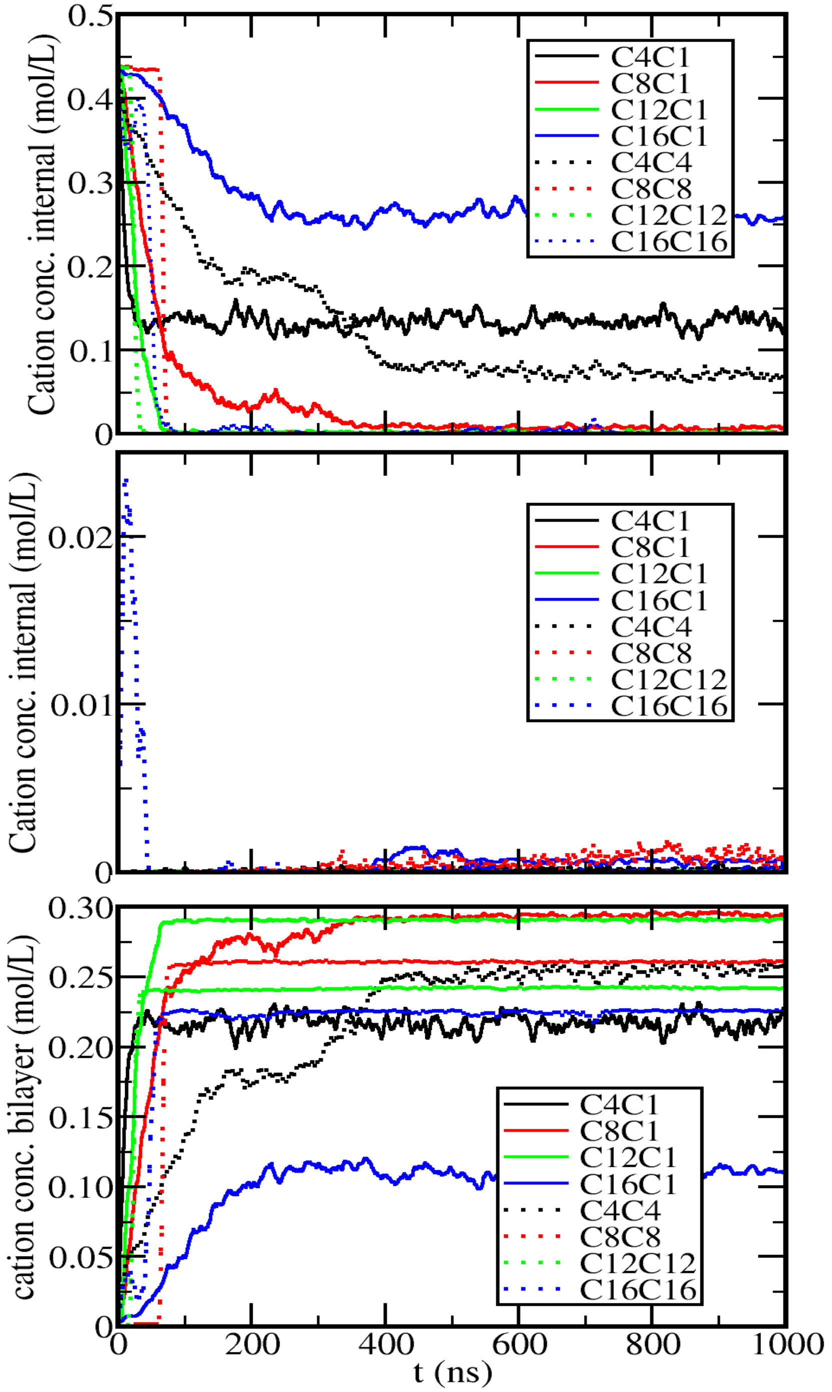

Figure S24 – Dynamics of cation penetration - cation concentration in external solution (top), internal solution (middle) and inside the lipid bilayers (bottom) along the simulations. A running average was performed at every 12 frames interval to reduce the noise.
